# Evolution as a result of resource flow in ecosystems: Ecological dynamics can drive evolution

**DOI:** 10.1371/journal.pone.0286922

**Published:** 2023-10-05

**Authors:** Mohammad Salahshour

**Affiliations:** 1 Max Planck Institute for Mathematics in the Sciences, Leipzig, Germany; 2 Max Planck Institute of Animal Behavior, Radolfzell, Germany; 3 Centre for the Advanced Study of Collective Behaviour, University of Konstanz, Konstanz, Germany; 4 Department of Biology, University of Konstanz, Konstanz, Germany; Teesside University, UNITED KINGDOM

## Abstract

To see how the flow of energy across ecosystems can derive evolution, I introduce a framework in which individuals interact with their peers and environment to accumulate resources, and use the resources to pay for their metabolic costs, grow and reproduce. I show that two conservation principles determine the system’s equilibrium state: conservation of resources- a physical principle stating that in the equilibrium, resource production and consumption should balance, and payoff equality- an economic principle, stating that the payoffs of different types in equilibrium should equal. Besides the equilibrium state, the system shows non-equilibrium fluctuations derived by the exponential growth of the individuals in which the payoff equality principle does not hold. A simple gradient-ascend dynamical mean-field equation predicts the onset of non-equilibrium fluctuations. As an example, I study the evolution of cooperation in public goods games. In both mixed and structured populations, cooperation evolves naturally in resource-poor environments but not in resource-rich environments. Population viscosity facilitates cooperation in poor environments but can be detrimental to cooperation in rich environments. In addition, cooperators and defectors show different life-history strategies: Cooperators live shorter lives and reproduce more than defectors. Both population structure and, more significantly, population viscosity reduce lifespan and life history differences between cooperators and defectors.

## Introduction

Throughout the history of life on earth, the production, consumption, and flow of energy across ecosystems have given rise to the evolution of complex forms of life and social structure [1, 2]. Natural selection, together with the concept of fitness, has remained core to our effort to understand this complexity. Much of the mathematical literature on evolutionary theory integrate these two concepts by trying to show how fitness-based selection can derive biological evolution [3–8], and generally bypass the conceptual and empirical difficulties confronting fitness-based selection [7–12], by postulating fitness as a quantitative determinant of evolutionary success [7, 8]. In evolutionary game theory, for instance, a Darwinian view of evolution is formalized by postulating fitness as a function of individuals’ relative payoff and requiring individuals to be selected for reproduction with a probability proportional to their fitness using different population dynamics, such as replicator dynamics [3–6, 13–15]. This framework has been commonly employed in evolutionary game theory and population genetic models and has successfully explained many evolutionary phenomena [3–5, 7, 8, 15–21]. However, conventional models of evolution appear to be only useful abstractions, as in the physical world evolution occurs as a result of organisms’ energy acquisition and production, energy consumption, growth and reproduction, and arguably, selection can occur either indirectly and as a result of organisms’ growth and reproduction, or merely as a useful abstraction. This observation raises the question of how individuals in populations evolve as a result of production, consumption, and flow of resources in ecosystems? To address this question, here I introduce an agent-based model in which individuals interact with their peers via a game and interact with the environment by accumulating environmental resources, and use these resources to pay for their metabolic functions, and to reproduce. In analogy with statistical physics, the equilibrium state of such an eco-evolutionary system can be described using two conservation principles: conservation of resources, which is a physical principle stating that resource production and resource consumption should balance in equilibrium, and payoff equality, which is an economic principle stating that in equilibrium the payoffs of coexisting types should equal. Besides equilibrium, the system shows non-equilibrium fluctuations derived by the exponential growth of organisms which derives the system out of economic equilibrium. A simple gradient ascend dynamical mean-field equation accounts for the equilibrium state and predicts the break-down of the equilibrium physics and the onset of the non-equilibrium fluctuation. The framework is also a natural setting to study life-history and reproduction strategies [18, 22, 23]. These observations reveal how ecological processes governing flow, production, and resource use in ecosystems can govern evolutionary phenomena, and how the eco-evolutionary dynamics can be grasped using similar principles to those governing physical systems [1, 2, 24, 25].

While the framework can readily be used to consider any interaction, as an example, I study the evolution of cooperation in public goods game, a classic problem in evolutionary game theory [20, 26–30] and experimental economics [31–33] and of interest in a wide range of issues from strategic interactions between animals and humans [15, 26–28, 31, 34–37] to common resource management [38, 39]. I show that the evolution of cooperation is no longer a puzzle in this framework. The model predicts that in both well-mixed and structured populations cooperation evolves in resource-poor environments but not in resource-rich environments. Non-equilibrium fluctuations further favor the evolution of cooperation by separating cooperators and defectors in time and/or in space, resulting in fewer opportunities for defectors to exploit cooperators. In contrast to what is usually argued [15, 28, 34, 36], the model suggests that population viscosity can be detrimental for the evolution of cooperation due to resource competition. Analysis of the evolved life history and reproduction strategies of the individuals show that cooperators reproduce more and live shorter lives than do defectors. Besides, both population structure and population viscosity favor shorter lifespan and thus often higher population turn-over.

## Results

**Fig 1:**
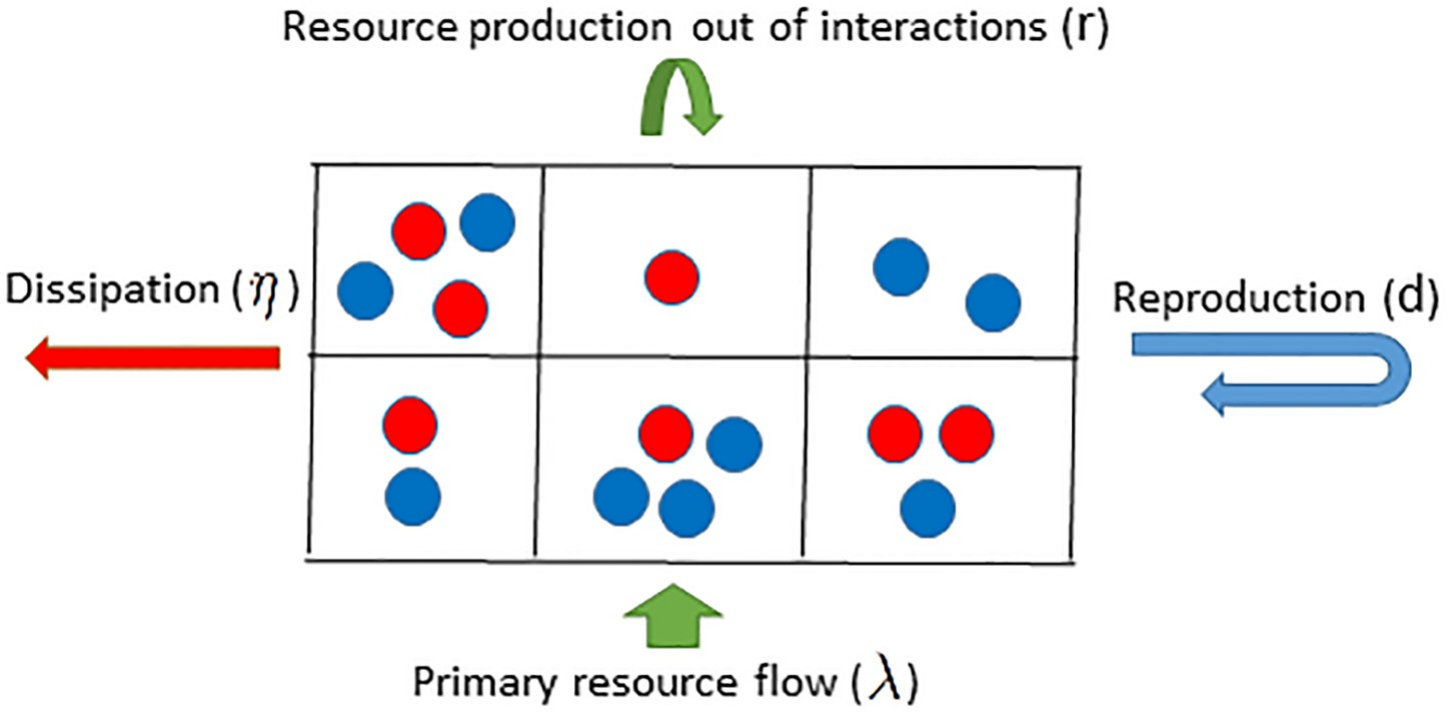
Evolution as a result of resource flow in ecosystems. Individuals gain energy due to the flow of primary resources (with a rate λ per lattice site) and resource production out of interactions, and dissipate energy due to their metabolic costs (with a rate *η* per individual). Individuals die if go out of energy and reproduce when their internal energy reaches a threshold *d*, and pass their strategy (color coded) to their offspring, subject to mutations. In the absence of mutations, the model reduces to a purely ecological model of resource flow and production, and in the presence of mutations, evolution results. The interaction between individuals can be of any forms, and is modeled here as a public goods game with enhancement factor *r*.

The model is presented in Fig 1. I consider an ecosystem in which individuals move and visit sites on a two-dimensional space, and obtain resources from two sources. There are basal resources, *κ*, in the environment which individuals can obtain. Basal resources can be considered as primary productivity and are regenerated by a constant rate λ per lattice site. In addition, individuals play a game and obtain payoffs, *π*, from the game, which is translated into a metabolic resource. Thus, each individual *α*, has internal energy, *ϵ*_*α*_, which evolves according to the following equation:

$$\begin{array}{c} \epsilon_{\alpha }\left(t\right)=\epsilon_{\alpha }\left(t-1\right)+\kappa_{\alpha }\left(t\right)+\pi_{\alpha }\left(t\right)-\eta \end{array}$$ (1)

Here, *κ*_*α*_ and *π*_*α*_ are, respectively, the basal resources and the payoff gained by individual *α*, and *η* is the resource consumption rate. Individuals die when their internal energy reaches zero and reproduce when their energy exceeds a threshold *d*. When reproducing, the individual breaks in half, and its energy is shared between the individual and its offspring. The offspring inherit the strategy of its parent subject to mutation, which occurs with probability *ν*. I assume individuals move at each time step with probability *q*, in which case the individual moves to a neighboring site. I consider two population structures: a lattice with Moore connectivity in which each site is connected to each of its eight neighbors and a mixed population in which all the sites are connected.

This framework is a general framework for evolution and the interactions between individuals can be of any form. Here, as an example, I consider a public goods game, a classic model of collective action problems [27–29, 31, 32]. Individuals play a public goods game with all the individuals present on the same grid site. Individuals can be cooperators or defectors. Cooperators pay a cost *c* to invest in a public resource. Defectors pay no cost and do not invest. All the investments are multiplied by an enhancement factor *r* > 1, reflecting the synergistic effect of cooperation, and are divided equally among the individuals. In the following, I denote the density of the individuals, cooperators, and defectors, respectively, as *ρ*, *ρ*_*C*_, and *ρ*_*D*_.

The system’s equilibrium state can be derived using two simple principles: resource conservation and payoff equality. Resource conservation is a physical principle and states that resource production and consumption should balance in equilibrium and can be stated as (*r* − 1)*cρ*_*C*_ + λ = *η*(*ρ*_*D*_ + *ρ*_*C*_). Payoff equality is an economic principle and dictates that in the equilibrium, the payoffs of all the non-extinct types should be equal, ${\bar{\pi }}_{C}+{\bar{\kappa }}_{C}={\bar{\pi }}_{D}+{\bar{\kappa }}_{D}$. ntuitively, this is due to the fact that an excess in recource production leads to population growth, and higher payoff of a subpopulation leads to the growth of that subpopulation. This intutition can be mathematically grasped by a simple gradient ascend equation derived in the Methods, by noting that the fixed point of the mean field equations are given by equilibrium conditions. In the derivation of the equilibrium solutions, I only consider perfect mixing (*q* = 1). As shown in Methods, using the two principles, the equilibrium densities can be derived as follows:

$$\begin{array}{c} \left\{ \begin{array}{cc} \rho_{D}^{I}=\lambda /\eta ,\;\rho_{C}^{I}=0\; & if\;\rho_{C}^{II}<0\\ \rho_{D}^{II}=r+W_{0}\left[-r\;\text{exp}\;\left(-r\right)\right]-\rho_{C}^{II},\;\rho_{C}^{II}=\frac{-\lambda +r\eta +\eta W_{0}\left[-r\;\text{exp}\;\left(-r\right)\right]}{c(r-1)}\; & if\;\rho_{C}^{II},\rho_{D}^{II}>0\\ \rho_{D}^{III}=0,\;\rho_{C}^{III}=\frac{\lambda }{\eta -(r-1)c}\; & if\;\rho_{D}^{II}<0 \end{array}\right. \end{array}$$ (2)

Here, *W*_0_ is the Lambert *W* function. The first solution describes a phase where cooperators do not survive, the second solution describes a phase where cooperators and defectors coexist in equilibrium and the last one gives a fully cooperative phase. As will be explained shortly, the condition $\rho_{C}^{II}<0$ can be solved analytically and gives a condition for the evolution of cooperation.

**Fig 2:**
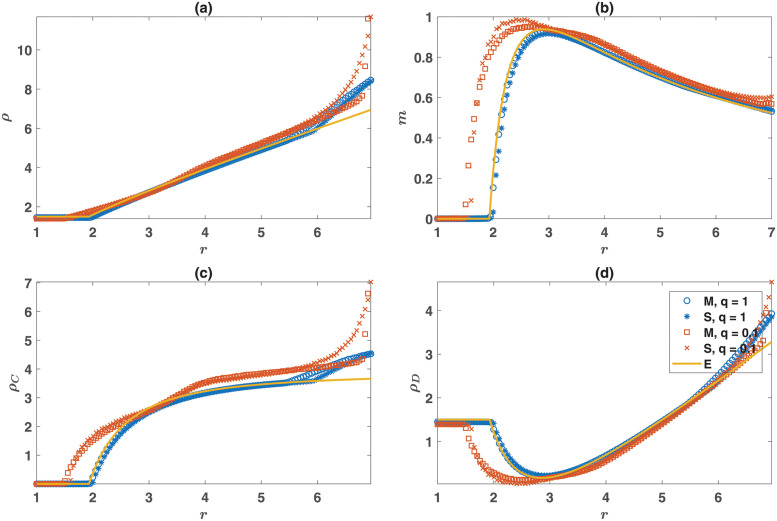
Densities of strategies. The density, *ρ*, (a), fraction of cooperators, *m*, (b), the density of cooperators, *ρ*_*C*_, (c), and the density of defectors, *ρ*_*D*_, (d), as a function of the enhancement factor, *r*. The equilibrium prediction, Eq 2 is shown by solid orange line. Simulations in non-viscous populations in both mixed and structured populations (blue circle and stars) show a high agreement with equilibrium prediction up to onset of non-equilibrium fluctuation for large enhancement factors. Parameter values: *L* = 100, *η* = 0.4, *d* = 2, λ = 0.6, *c* = 0.1, and *ν* = 10^−4^. M denotes mixed population, S structured population, and E denotes equilibrium prediction.

The equilibrium prediction Eq (2), describes the stationary state of the system with a high agreement with simulation results for small values of *r* where the system settles in a fixed point (see Fig 2 for dependence on *r* and the S1 Fig in S1 Text for dependence on λ). For larger *r* the system shows periodic fluctuations in a well-mixed population. These fluctuations are evident as spatio-temporal traveling waves in a structured population (see the S1–S4 Videos and S2–S7 Figs in S1 Text). In the non-equilibrium state, payoffs do not equal, and the system does not reach economic equilibrium. Instead, game payoffs, basal resources, and densities of cooperators and defectors fluctuate. Non-equilibrium fluctuations lead to temporal or spatio-temporal separation of cooperators and defectors, which leads to fewer opportunities for defectors to exploit cooperators. This separation allows, at times, a higher fraction of cooperators, and thus, a higher resource production than that predicted by the payoff equality principle. Consequently, the time average density of individuals, cooperators, and defectors all overshoot the equilibrium value.

The onset of non-equilibrium fluctuations can be grasped by a simple dynamical mean-filed equation based on the idea that the growth rate of different types is proportional to their net resources:

$$\begin{array}{cc} & \frac{d}{dt}\rho_{C}={\bar{\pi }}_{C}+\lambda /\left(\rho_{C}+\rho_{D}\right)-\eta ,\\ & \frac{d}{dt}\rho_{D}={\bar{\pi }}_{D}+\lambda /\left(\rho_{C}+\rho_{D}\right)-\eta . \end{array}$$ (3)

Where, ${\bar{\pi }}_{C}$ and ${\bar{\pi }}_{D}$ are the average payoffs of cooperators and defectors (See Methods and the Supplementary Note. 4 in S1 Text). This simple dynamical equation predicts the behavior of the model fairly well. Particularly, the dynamic has two boundary fixed points corresponding to solutions *I* and *III*, and an interior fixed point corresponding to solution *II*. The interior fixed point becomes unstable at a critical value of *r*, giving rise to non-equilibrium fluctuations (see the S8 and S9(a) Figs in S1 Text)

**Fig 3:**
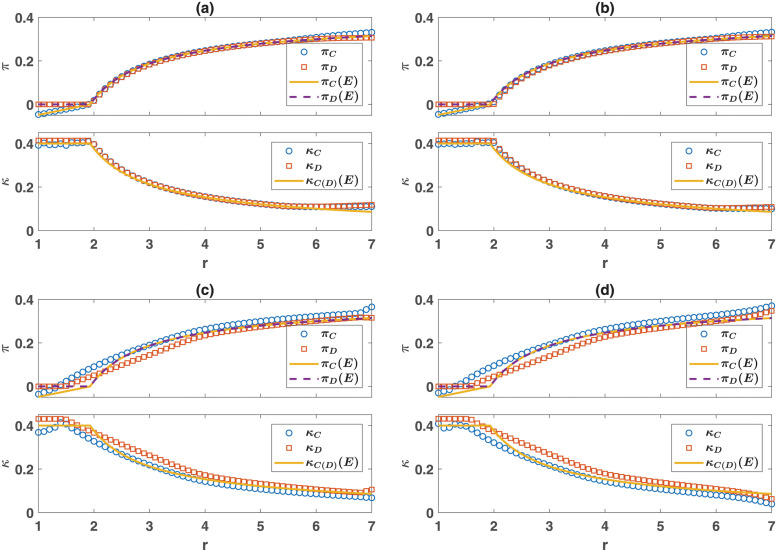
Payoffs and resource acquisition. Game payoffs (top) and basal resource acquisition (bottom) of cooperators (blue circles) and defectors (red squares). Equilibrium predictions for payoffs of cooperators (solid orange line) and defectors (dashed purple line) are also shown (top). The equilibrium prediction for basal resource acquisition for cooperators and defectors is the same and is shown by a solid orange line (bottom). In (a) a mixed non-viscous (*q* = 1) population, in (b) a structured non-viscous population, in (c) a viscous (*q* = 0.1) mixed population, and in (d) a structured viscous population is shown. While in a non-viscous population, payoffs and basal resource acquisition of cooperators and defectors are the same, in a viscous population, cooperators obtain a higher game payoff and a lower basal resource acquisition due to the formation of homogeneous domains. Parameter values: *L* = 100, *η* = 0.4, *d* = 2, λ = 0.6, *c* = 0.1, and *ν* = 10^−4^.

Examination of the case of *q* = 0.1 in Fig 2 shows that population viscosity has contrasting effects on individuals’ resource acquisition. While in non-viscous populations, cooperators and defectors receive the same game payoff and basal resources, which is well predicted by the equilibrium solution (Fig 3(a) and 3(b)), the situation is different in a viscous population. Viscosity leads to the formation of homogeneous blocks of cooperators, within which cooperators can reach a higher game payoff than defectors. However, the excess payoff escalates resource competition: Cooperators receive a lower per-capita basal resource compared to defectors due to living in over-crowded domains (Fig 3(c) and 3(d)). This trade-off raises the question of whether population viscosity can benefit cooperation? As Fig 2 suggests, this is the case in poor environments, as the evolution of cooperation occurs for a smaller enhancement factor in a viscous population. However, as we will shortly see, this is not the case in rich environments.

**Fig 4:**
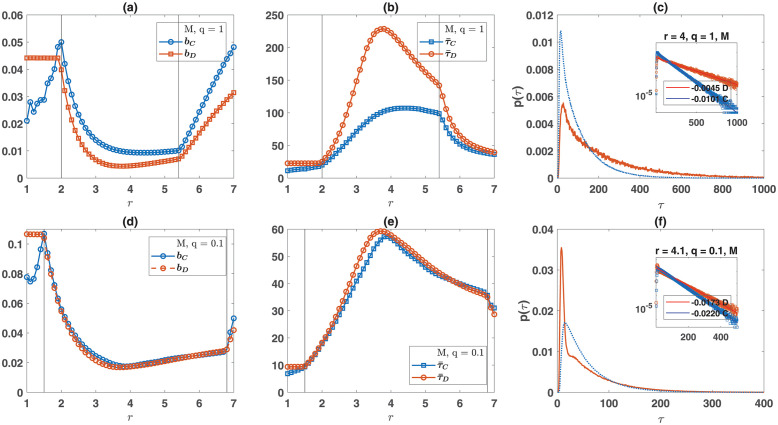
Life-histories. The mean birth rate of cooperators, *b*_*C*_, and defectors, *b*_*D*_, for a non-viscous (a), and viscous (d) population, the mean lifespan of cooperators, ${\bar{\tau }}_{C}$, and defectors, ${\bar{\tau }}_{D}$, for a non-viscous (b), and viscous (e) population, and the lifespan distribution of cooperators (solid blue) and defectors (dotted red) for a non-viscous (c), and viscous (f) population are shown. Lifespan distributions show a characteristic age with an exponential cut-off, which is faster for cooperators. Cooperators and defectors show different life-histories: cooperators reproduce more and live shorter. Population viscosity decreases lifespan and tends to diminish the difference in life-histories. In a viscous population, the lifespan distribution of defectors shows a peak at early ages, resulting from the rapid demise of defectors once they drive cooperators in their neighborhood o extinction. Parameter values: *L* = 100, *η* = 0.4, *d* = 2, λ = 0.6, *c* = 0.1, and *ν* = 10^−4^. In (a) and (b) *q* = 1 and in (d) and (e) *q* = 0.1. In (c) and (f) *r* = 4. A mixed population is used.

An interesting prediction of the model is that cooperators have a higher population turn-over, i.e., a higher birth and mortality rate. This is due to the fact that cooperators are resource producers which makes them vulnerable to exploitation when accompanied by defectors. Examination of the time average birth rate of cooperators and defectors plotted as a function of *r* in Fig 4(a), shows this is the case in all the region where cooperators survive. A higher population turn-over of cooperators suggests that cooperators have a shorter lifespan. This can be seen to be the case in Fig 4(b), and shows that cooperators and defectors have different life history strategies: cooperators live shorter lives and reproduce more while defectors live longer lives and reproduce less. Furthermore, the onset of non-equilibrium fluctuations is accompanied by a decline in the average lifespan of the individuals. A closer look into the age distribution of cooperators and defectors, plotted in Fig 4(c), reveals cooperators and defectors have a characteristic lifespan with an exponential decay at long ages, which is faster for cooperators (see the S10 Fig in S1 Text for the distribution of lifespans in the non-equilibrium phase).

Population viscosity tends to equalize the population turn-over and average lifespan of cooperators and defectors (Fig 4(d) to 4(f)). Furthermore, the lifespan distribution of defectors shows a peak at young ages in viscous populations due to the fact that lower mobility restricts defectors’ ability to exploit cooperators, such that mutant defectors rapidly die out once they drive cooperators in their neighborhood to extinction. Importantly, population viscosity decreases the average lifespan. Besides, the lifespan distribution of individuals in a viscous population shows a faster decay for higher ages (compare Fig 4(c) and 4(f)). That is, in viscous populations, individuals show a different life-history strategy: they live shorter lives and reproduce more. This result, to a smaller degree, holds in structured populations (see S11 and S12 Figs in S1 Text).

**Fig 5:**
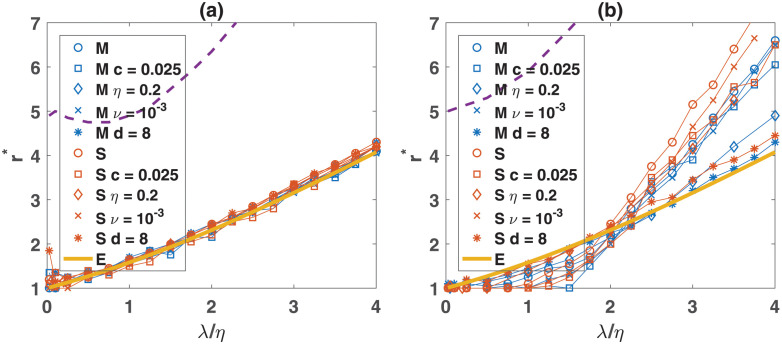
Phase transitions. The phase diagram of the model in *r*−λ/*η* plane for a non-viscous (a) and viscous (b) population. Markers show simulation results for different parameter values. The solid orange line shows the equilibrium prediction of the transition to cooperative phase. Dashed lines show the onset of non-equilibrium fluctuations. Consistently with simulation results, the equilibrium argument predicts in a non-viscous population this transition depends only on λ/*η* and is independent of other parameter values. The base parameter values are *d* = 2, *ν* = 10^−3^, *c* = 0.1, *L* = 100.

Using the conservation principles, it is also possible to derive expressions for the onset of cooperation. As shown in Methods, for non-zero primary resource production, the payoff equality principle predicts that the onset of cooperation occurs at an enhancement factor equal to $r^{*}=\frac{\lambda /\eta }{1-\text{exp}(-\lambda /\eta )}$. I note that, this condition also results from $rho_{C}^{II}=0$. For zero primary productivity, the resource conservation principle implies that cooperation evolves when *r* > 1+ *η*/*c* (see the S9(b) Fig in S1 Text). In Fig 5(a) (non-viscous) and Fig 5(b) (viscous), the phase diagram of the system in *r*−λ/*η* plane for different parameter values is plotted by different marker types (see the S13 and S14 Figs in S1 Text for the densities of strategies). Consistently with the prediction of payoff equality principle (orange solid line), the transition to the evolution of cooperation only depends on λ/*η*, is independent of other parameter values, and remarkably collapses into the equilibrium curve in a non-viscous population when plotted as a function of λ/*η*. This shows te evolution of cooperation is solely determined by primary productivity and resource consumption.

Examination of the phase diagrams shows that cooperation evolves in poor environments, and as λ increases a transition to a phase where cooperation does not evolve occurs. Interestingly, the densities and birth rates of cooperators is maximized at the transition point (Fig 4(a) and 4(d), and S15 and S16 Figs and S1 Text). Furthermore, while for small resource regeneration rate population viscosity facilitates the evolution of cooperation, for large resource regeneration rate, population viscosity hinders the evolution of cooperation by shifting the transition to larger enhancement factors (see the S17 Fig in S1 Text for the quantitative difference between viscous and non-viscous populations).

## Discussion

As we have seen, the flow, production, and consumption of energy across ecosystems provides a natural framework for evolutionary processes. By showing that such a model of evolution can exhibit both equilibrium states, which can be described by resource conservation and payoff equality principles, and non-equilibrium states, in which payoff equality is violated due to the exponential growth of the individuals, this framework reveals that, as it has been called for by some arguments [1, 2, 24, 25], evolutionary phenomena has fundamental similarities with physical systems and can be fruitfully studied using similar principles to those used to study physical and economic systems. The model introduced here also emphasizes the ecological nature of evolutionary phenomena. In this view, this framework reveals how the flow, production, and consumption of resources in ecosystems can derive evolutionary processes and accounts for the high level of complexity and organization produced in the course of evolution. I note that, the framework studied here, in the absence of mutations (*ν* = 0) and possibly in the presence of immigration, can be regarded as a purely ecological model. Such a purely ecological model has recently been used to study predator-prey interactions and grasps fundamental laws of ecological systems [40]. This observation suggests modeling evolutionary process based on the flow of resources may be a key to understanding fundamental laws of eco-evolutionary systems.

Using public goods game as an example of the interaction, I showed that cooperation evolves in poor environments, but not in rich environments. In a non-viscous population, cooperation evolves due to density fluctuations: While cooperators always receive a lower payoff compared to defectors in their site, the expected payoff of cooperators can be larger than those of defectors due to density fluctuations. This is an instance of Simpson’s paradox which is noted to underlie the evolution of cooperation in voluntary games [41, 42], or interacting public goods games [35, 43, 44]. In a non-viscous population, cooperation can be further bolstered by population viscosity, a well-known pathway to the evolution of cooperation [15, 28, 34, 36]. However, surprisingly, population viscosity can be detrimental for the evolution of cooperation in resource-rich environments. This suggests population viscosity, or alternatively mobility, which is argued to be sometimes beneficial for the evolution of cooperation [45–47], can have contrasting effects depending on resource availability. The framework also allows to study life-history strategies, a factor that has remained largely unexplored in the theoretical studies on the evolution of cooperation, with the possible exception of cooperative breeding [48, 49]. The model shows that by individuals balancing their resource acquisition and consumption, natural cooperative and defective life histories emerge [10] and both population viscosity and population structure decrease the individuals’ lifespan and life-history differences between cooperators and defectors.

The framework developed here could also have implications for social evolution. Just as biological evolution proceeds due to energy use, growth and reproduction of organisms, economic, cultural, or social evolution, proceed by the growth of successful entities [25, 50], and the similarity between the two is widely noted [3, 17, 51]. The eco-evolutionary model of evolution introduced here provides a unified framework to study evolution of entities as a result of organism’s resource use and growth. In this regards, applying this framework to public goods game already sheds light on the the physical principles governing the equilibrium and non-equilibrium eco-evolutionary dynamics of a simple evolutionary-economic model of resource production and use.

The framework introduced here can also shed light on the current socio-ecological challenges. The global ecosystem is facing numerous challenges, such as biodiversity loss [52, 53], climate change [54, 55], and overuse of public resources [55, 56], that are putting it under increasing pressure [38, 39]. Past research has pointed out the necessity of theoretical research to better understand these challenges by focusing on the socio-environmental consequences of human behavior. An important question to be addressed in this regard is how the individuals’ and populations’ behavior may affect the production and flow of resources into the ecosystem. The framework introduced here can be of interest in addressing this question in future research by focusing on the eco-evolutionary consequences of strategic interactions.

## Methods

### Overview of the model and simulations

Simulations of the model are performed based on the model definition. Unless otherwise stated, the simulations start from an initial condition in which all the individuals are defectors and their initial lattice site is chosen independently at random. The simulations start with 100 individuals. Since the model does not show bistability, the same stationary state is reached starting from other initial conditions. In the simulations, all the individuals first gather basal resources and gain payoffs from playing the game. Basal resources available on a site are divided equally among all the individuals visiting that site. Game payoffs are attributed to the individuals based on the public goods game they play. That is, cooperators, pay a cost *c* to invest the same amount in a public resource, and defectors pay no cost and do not invest. All the investments are multiplied by an enhancement factor *r* and are divided equally among the individuals. After this stage, individuals reproduce. In the reproduction stage, all the individuals whose internal resource is above the threshold *d* reproduce an offspring. The offspring inherits the strategy of its parent subject to mutation. Mutations occur with probability *ν*. In the case of a mutation, the strategy of the offspring is flipped to its opposite value (*C* to *D* and vice versa). The parent’s resource is divided equally among the parent and the offspring. Individuals move into a new lattice site after reproduction. In the movement stage, each individual moves into a neighboring lattice site with probability *q*. In the mixed population, all the lattice sites are connected. Thus individuals can jump into any new location. For the structured population, a lattice with Moore connectivity is considered in which each site is connected to eight neighbors to its top, bottom, left, and right. In the last stage, individuals pay the metabolic cost, and then those whose internal resource goes below zero die out of the population. In the case that the population goes to extinction, assuming there is immigration, an individual with a random strategy is added to the population. I note that extinction can occur only for a resource regeneration rate close to zero and in a non-viscous population. In the last stage, the resource in each site increase by an amount λ.

I note that in the model considered here, the individuals receive the total payoff of the game, that is, the benefit minus cost, before reproduction and death. In realistic contexts, this can be relevant when the time scale of playing the game is short compared to generation time or when the cost and benefit of the game are paid and accrued gradually. Examples can be public good production when the cost of production and the benefit of acquiring the public good occur simultaneously or continuously, such as siderophore production in bacteria [43], cooperative hunting [57, 58], and anti-predator behavior such as alarm calls [59]. In contexts where there is a delay between paying the cost and accruing benefit, it can be more relevant to introduce a separation between the two events and allow individuals to die if their internal resource drops below zero in the investment stage. This can make the resource gain by cooperators dependent on their internal resources and leads to corrections in the total resource gain dependent of *d*, *c*. However, these corrections are not expected to lead to significant differences when *c* is much smaller than *d*.

### Statistics and reproducibility

Simulations in Fig 2 in are performed for *T* = 10000 time steps for a non-viscous and *T* = 5000 time steps for a viscous population, and a time average is taken after time 4000. In Figs 3 and 4, the same set of simulations are used. These simulations are run for 10000 time steps. The time average in Fig 3 is performed after time 3000. The lifespan distribution is calculated after time 3000, where stationarity is ensured. In Fig 5, panels (a) and (b), different sets of simulations for different parameter values are used to determine the phase boundaries. To determine the onset of non-equilibrium fluctuations in Fig 5, the mean-field equation is numerically solved for different values of cost. Simulations used in Fig 5 are run for 15000 time steps, and time averages are taken after the first 2000 time steps.

### Analytical results: Derivation of equilibrium densities

The system’s equilibrium state can be derived using two principles, resource conservation, according to which the resource production and consumption should balance in the stationary state and payoff equality, according to which the payoff of all the surviving types should be equal:

$$\begin{array}{c} Resource\;conservation:\;\text{resources}\;\text{produced}=\text{resources}\;\text{consummed},\\ Payoff\;equality:\;\;{\bar{\pi }}_{C}+{\bar{\kappa }}_{C}={\bar{\pi }}_{D}+{\bar{\kappa }}_{D}. \end{array}$$ (4)

In the following, in the derivation of the equilibrium solutions, I only consider perfect mixing (*q* = 1). In this case, ${\bar{\kappa }}_{C}={\bar{\kappa }}_{D}=\lambda /\rho$, and payoff equality equation reads as ${\bar{\pi }}_{C}={\bar{\pi }}_{D}$. Resources are produced due to two sources: the flow of basal resource into the system at a rate λ per site and resource production by cooperators, (*r* − 1)*cρ*_*C*_. Resources are consumed by a rate *η*(*ρ*_*D*_ + *ρ*_*C*_). Thus, we have for the resource conservation equation:

$$\begin{array}{c} \left(r-1\right)c\rho_{C}+\lambda =\eta \left(\rho_{D}+\rho_{C}\right). \end{array}$$ (5)

To write an explicit equation for the payoff equality, we need to calculate the payoff of cooperators and defectors. Noting that the payoff of a cooperator with *n*_*C*_ cooperators and *n*_*D*_ defectors in its site is equal to $rc\frac{1+n_{C}}{1+n_{C}+n_{D}}-c$, and the payoff of a defector is equal to $rc\frac{n_{D}}{1+n_{C}+n_{D}}$), and the probability that a cooperator or defector finds *n*_*s*_ individuals of type *s* in its site is governed by the Poisson distribution, the average payoffs of cooperators and defectors can be calculated as follows:

$$\begin{array}{cc} & {\bar{\pi }}_{C}=\sum_{n_{C}=0}^{\infty }\sum_{n_{D}=0}^{\infty }\left[rc\frac{1+n_{C}}{1+n_{C}+n_{D}}-c\right]\frac{n_{C}^{\rho_{C}}}{n_{C}!}\;\text{exp}\;\left(-\rho_{C}\right)\frac{n_{D}^{\rho_{D}}}{n_{D}!}\;\text{exp}\;\left(-\rho_{D}\right)=\\ & \;-\frac{c(\left(1-r\right)\rho_{C}^{2}+\left(2-r\right)\rho_{C}\rho_{D}+\rho_{D}\left(r\left(\text{exp}\;\left(-\rho_{D}-\rho_{C}\right)-1\right)+\rho_{D}\right)}{{\left(\rho_{C}+\rho_{D}\right)}^{2}},\\ & {\bar{\pi }}_{D}=\sum_{n_{C}=0}^{\infty }\sum_{n_{D}=0}^{\infty }rc\frac{n_{D}}{1+n_{C}+n_{D}}\frac{n_{C}^{\rho_{C}}}{n_{C}!}\;\text{exp}\;\left(-\rho_{C}\right)\frac{n_{D}^{\rho_{D}}}{n_{D}!}\;\text{exp}\;\left(-\rho_{D}\right)=\\ & \frac{c\;\text{exp}\;\left(-\rho_{C}-\rho_{D}\right)r\rho_{C}\left(1+\;\text{exp}\;\left(\rho_{C}+\rho_{D}\right)\left(-1+\rho_{C}+\rho_{D}\right)\right)}{{\left(\rho_{C}+\rho_{D}\right)}^{2}}. \end{array}$$ (6)

I note that these expressions hold only in the case of perfect mixing, *q* = 1. Limited dispersal (smaller *q*) leads to assortative interactions. That is, the probability that *n*_*C*_ cooperators to be found in a site occupied by a focal cooperator are different from that for a focal defector for smaller values of *q*. Using Eq 6 in Eq 4, we have for the payoff equality equation:

$$\begin{array}{cc} & -\frac{c(\left(1-r\right)\rho_{C}^{2}+\left(2-r\right)\rho_{C}\rho_{D}+\rho_{D}\left(r\left(\;\text{exp}\;\left(-\rho_{D}-\rho_{C}\right)-1\right)+\rho_{D}\right)}{{\left(\rho_{C}+\rho_{D}\right)}^{2}}=\\ & \frac{c\;\text{exp}\;\left(-\rho_{C}-\rho_{D}\right)r\rho_{C}\left(1+\;\text{exp}\;\left(\rho_{C}+\rho_{D}\right)\left(-1+\rho_{C}+\rho_{D}\right)\right)}{{\left(\rho_{C}+\rho_{D}\right)}^{2}} \end{array}$$ (7)

Eqs 7 and 5 give a set of two equations in *ρ*_*C*_ and *ρ*_*D*_ which can be solved to derive equilibrium densities:

$$\begin{array}{c} \rho_{C}^{II}=\frac{-\lambda +r\eta +\eta W_{0}\left[-r\;\text{exp}\;\left(-r\right)\right]}{c(r-1)}\;\;\rho_{D}^{II}=r+W_{0}\left[-r\;\text{exp}\;\left(-r\right)\right]-\rho_{C}^{II} \end{array}$$ (8)

Where *W*_0_ is the Lambert W function. I note that the argument leading to Eq 7 holds only if cooperators and defectors coexist and should be complemented by proper expressions for the cases that one of the two goes to extinction. When *ρ*_*C*_ = 0, using Eq 5, the equilibrium density of defectors is given by *ρ*_*D*_ = λ/*η*, and when *ρ*_*D*_ = 0, Eq 5 reads as *ρ*_*C*_(*r* − 1)*c* + λ = *ρ*_*C*_*η*, which gives $\rho_{C}=\frac{\lambda }{\eta -(r-1)c}$. Thus we find the complete set of equilibrium densities, given in Eq 2.

### Conditions for the evolution of cooperation

It is possible to derive the necessary and sufficient conditions for the evolution of cooperation by using conservation principles. To do this, I note that the population is composed of all defectors at the onset of cooperation. Thus, the expected payoff of a defector from the game is zero. The expected payoff of a mutant cooperator can be calculated from the expressions in Eq 6 by noting that *ρ*_*C*_ = 0 in this case. Thus, the payoff equality equation at the onset of cooperation reads as:

$$\begin{array}{c} \sum_{n_{D}=0}^{\infty }\left[\frac{r^{*}c}{1+n_{D}}-c\right]\left(\frac{n_{D}^{\rho_{D}}}{n_{D}!}\;\text{exp}\;\left(-\rho_{D}\right)\right)=0 \end{array}$$ (9)

From Eq 9, and using the fact that at the transition we have *ρ*_*D*_ = λ/*η*, we find for the transition point:

$$\begin{array}{c} r^{*}=\frac{\lambda /\eta }{1-\;\text{exp}\;(-\lambda /\eta )} \end{array}$$ (10)

This equation gives the necessary and sufficient condition for the evolution of cooperation. Remarkably, the evolution of cooperation is determined only by basal resource generation λ and resource consumption rate, *η*, and is independent of other parameters of the model. In other words, the evolution of cooperation is determined only by the flow of resources into the system and resource dissipation by consumption. Intuitively, this is due to the fact that evolution of cooperation is determined by the densities of individuals, and equilibrium densities at the onset of cooperation, in turn, are determined by resource production (λ) and resource consumption (*η*). Eq 10 holds only for non-zero basal resource generation rate. It is possible to derive the condition for the evolution of cooperation for zero basal resource generation rate as well. To do this, I note that by dividing the resource conservation equation by *ρ*_*D*_, we have for the fraction of cooperators to defectors, *γ*:

$$\begin{array}{c} \gamma =-\frac{\lambda }{\rho_{D}\left[\left(r-1\right)c-\eta \right]}+\frac{\eta }{(r-1)c-\eta }. \end{array}$$ (11)

In the limit of zero external resources, this reads:

$$\begin{array}{c} \gamma =\frac{\eta }{(r-1)c-\eta }. \end{array}$$ (12)

By imposing the condition *γ* > 0, we find for the condition for the evolution of cooperation in this limit:

$$\begin{array}{c} r^{*}>1+\eta /c. \end{array}$$ (13)

That is, the surplus of cooperation (*c*(*r* − 1)) should be larger than the resource consumption rate *η*.

### Mean-filed equations

A dynamical mean-field equation can be written for the model based on the idea that the growth of cooperators and defectors is proportional to their net resources, that is a subpopulation *x* ∈ *C*, *D*, grows if the average resource gain per individual, ${\bar{\pi }}_{x}+\lambda /\left(\rho_{C}+\rho_{D}\right)$, is larger than its resource consumption rate, and declines otherwise:

$$\begin{array}{cc} & \frac{d}{dt}\rho_{C}={\bar{\pi }}_{C}+\lambda /\left(\rho_{C}+\rho_{D}\right)-\eta ,\\ & \frac{d}{dt}\rho_{D}={\bar{\pi }}_{D}+\lambda /\left(\rho_{C}+\rho_{D}\right)-\eta . \end{array}$$ (14)

It is possible to amend this equation by including mutation rate, as follows:

$$\begin{array}{cc} & \frac{d}{dt}\rho_{C}=\left(1-\nu \right)\left[{\bar{\pi }}_{C}+\lambda /\left(\rho_{C}+\rho_{D}\right)-\eta \right]+\nu \left[{\bar{\pi }}_{D}+\lambda /\left(\rho_{C}+\rho_{D}\right)-\eta \right],\\ & \frac{d}{dt}\rho_{D}=\left(1-\nu \right)\left[{\bar{\pi }}_{D}+\lambda /\left(\rho_{C}+\rho_{D}\right)-\eta \right]+\nu \left[{\bar{\pi }}_{C}+\lambda /\left(\rho_{C}+\rho_{D}\right)-\eta \right]. \end{array}$$ (15)

Intutively, this equation can be justified by nothing that on average, a fraction 1 − *ν* of cooperators (defectors) mutate into defectors (cooperators). Both equations predict the existence of both equilibrium and non-equilibrium regimes. Namely, depending on the parameter values, these equations have two boundary fixed points corresponding to solutions *I* and *III*, where either only cooperators or defectors survive, and a coexistence fixed point, corresponding to solution *II*, where cooperators and defectors coexist. As seen in the Results Section, these solutions are in high agreement with simulation results for a non-viscous population in both well-mixed and structured populations. The mean-field equation also predicts the onset of non-equilibrium fluctuations: As the enhancement factors increase, the interior fixed point becomes unstable, and periodic fluctuations are observed (see the Supplementary Note 4 in S1 Text).

## Supporting information

S1 Text Supplementary analysis of the model. (PDF)

S1 Video Illustration of the dynamics of the model in the equilibrium state. (AVI)

S2 Video Illustration of the dynamics of the model in a nonequilibrium regime. (AVI)

S3 Video Illustration of the dynamics of the model in a nonequilibrium regime. (AVI)

S4 Video Illustration of the dynamics of the model in a nonequilibrium regime. (AVI)
