# Supplementary material for: Evolution as a result of resource flow in ecosystems: Ecological dynamics can drive evolution
Source: PLoS One. 2023 Oct 5;18(10):e0286922. doi: 10.1371/journal.pone.0286922 (PMC10553275; doi:10.1371/journal.pone.0286922)
Supplement: S1 Text — (PDF) [file pone.0286922.s001.pdf]

Supplementary Information for
Evolution as a result of resource flow in ecosystems: A novel framework for evolutionary processes

Contents

Supplementary Note.1 Phase transition as a function of resource regeneration rate, λ 1

Supplementary Note.2 Time evolution of the system 2

Supplementary Note.3 Supplementary Videos 3

Supplementary Note.4 Mean field equations 3

Supplementary Note.5 Phase transitions 3

Supplementary Note.6 Life-history strategies 4

Supplementary Note.7 The densities of different strategies 5

Supplementary Note.1 Phase transition as a function of resource regeneration rate, λ

The behavior of the system as a function of the resource regeneration rate λ is studied in Fig. Supplementary Figure.1. Here, the density of the individuals, ρ, the fraction of cooperators, $m = \rho_C/\rho$, the density of cooperators, ρ_C, and the density of defectors, ρ_D, are plotted. Simulation for non-viscous ($q = 1$) and viscous ($q = 0.1$), and structured and mixed populations are shown with different marker types, as indicated in the legend. In Supplementary Figure.1(a) to (d), $\eta = 0.4$ and in Supplementary Figure.1(e) to (h), $\eta = 0.1$.

The equilibrium solutions and simulations results show that the system shows that cooperation does not evolve for large enhancement factors, but it does evolve for small enhancement factors. Furthermore, the density of cooperators shows a peak at an optimal value of resource regeneration rate (c). This is the value of λ above which defectors survive, and the coexistence fixed point becomes stable. Quantitatively, simulation results for a non-viscous population show a high agreement with equilibrium solutions in the region where the dynamics settle in equilibrium. This is the case for large λ. For too small λ, the dynamics show periodic fluctuations. These fluctuations exhibit themselves in the form of periodic orbits in a mixed population and Spatio-temporal traveling waves in a sufficiently large structured population. In this region, economic equilibrium is not reached, and consequently, a deviation from equilibrium prediction is observed. I note that, in the fixed point, a small discrepancy between equilibrium solution and simulation results is expected for large η. This is due to the fact that those individuals who die out do not have exactly a zero internal resource but a negative internal resource, larger than $-\eta$. This introduces a deviation of the resource conservation equation proportional to the per capita death which is higher for larger η. As can be seen in Supplementary Figure.1, this deviation is at work for large $\eta = 0.4$, but a higher agreement between equilibrium predictions and simulation results is observed for smaller η.

The dynamic of the system is similar in a non-viscous population. In this case, non-equilibrium fluctuations are observed for small λ. The dynamics settle in a fixed point for large λ. Cooperators survive for small λ, and a transition to a phase where defectors survive is observed as λ increases. Comparison of viscous and non-viscous populations show that population viscosity facilitates the evolution of cooperation since the transition to the defective phase shifts to larger values of λ. However, as shown in the main text, this is the case only for small enhancement factors.

Supplementary Note.2 Time evolution of the system

The time evolution of the system is studied in Supplementary Figure.2 for a non-viscous and well-mixed population, in Supplementary Figure.3 for a non-viscous and structured population, in Supplementary Figure.4 for a viscous and mixed population, and in Supplementary Figure.5 for a viscous and structured population. In a non-viscous and well-mixed population, the system settles in a equilibrium where both resource conservation and payoff equality hold in small r, and shows periodic fluctuations where payoff equality does not hold for large r. In the non-equilibrium state, payoffs do not equal, and thus, the system does not reach economic equilibrium (Fig. Supplementary Figure.2(b), middle panel). Instead, game payoffs, π and basal resource, κ, of cooperators and defectors, as well as their densities, show periodic fluctuations. The dynamic in this regime is driven by bursts of proliferation of cooperators, with a higher than equilibrium density, payoff, and reproduction (Fig. Supplementary Figure.2(b), bottom panel), which are later replaced by defectors. Once defectors dominate, resource production is not sufficient to support the higher than equilibrium number of defectors, and thus, their density declines, paving the way for the invasion of cooperators in a second cycle. Non-equilibrium fluctuations lead to temporal or spatio-temporal separation of cooperators and defectors. This separation allows, at times, a higher fraction of cooperators, and thus, a higher resource production than that predicted by the payoff equality principle to be present in the system. As a result of this excess production resulting from the non-equilibrium assortment of cooperators, the time average density of individuals, cooperators, and defectors all overshoot the equilibrium value.

In a non-viscous and structured population, the system settles in a stationary state for small enhancement factors (Supplementary Figure.3(a)) and exhibit periodic fluctuations for large enhancement factors (Supplementary Figure.3(b)). In large system sizes, these fluctuations exhibit themselves in the form of Spatio-temporal traveling waves. Like a mixed population, cooperators show a higher population turnover than defectors in both states.

Similar states are observed in a viscous population: the system settles in a fixed point where economic equilibrium is reached, and total payoffs of cooperators and defectors are equal for small enhancement factors, and a non-equilibrium state where economic equilibrium is not reached and frequencies of cooperators and defectors show periodic fluctuations. These fluctuations exhibit themselves in the form of a periodic orbit in a mixed population and Spatio-temporal traveling waves in a structured population. Furthermore, due to population viscosity, cooperators receive a higher payoff from the game but a lower basal resource acquisition. This results from the fact that cooperators often live in compact homogeneous groups, in which cooperators reach the benefit of cooperation but obtain lower basal resources due to living in overcrowded domains. Finally, population viscosity tends to diminish the differences between cooperators' and defectors' population turnover in the equilibrium state. However, in the non-equilibrium fluctuating state in large enhancement factors, cooperators show a higher population turnover compared to defectors in both structured and mixed populations.

Supplementary Note.3 Supplementary Videos

The supplementary videos show the time evolution of the system in a structured population. The parameter values used in the videos are $d = 2$, $\nu = 10^{-2}$, $\eta = 0.4$, $r = 2$, $\lambda = 0.1$, $L = 100$. In SV.1 $c = 0.1$ and $q = 1$. A snapshot of the densities of the individuals in this regime is presented in Supplementary Figure.6(a). In Supplementary Figure.7(a), the theta function of the densities, $\theta(\rho(x, y))$ is presented. This function is equal to one only if $\rho(x, y)$ is larger than zero and it is zero otherwise. In this regime, the dynamics settle in an equilibrium state where economic equilibrium holds. This can be seen in the video, where starting from an initial condition where all the individuals are defectors, the dynamics rapidly settle in an equilibrium state with a high density of cooperators.

In SV.2, $c = 1$ and $q = 1$. A snapshot of the densities of the individuals in this regime is presented in Supplementary Figure.6(b). In Supplementary Figure.7(b), the theta function of the densities, $\theta(\rho(x, y))$ is presented. Here, the dynamics do not settle in equilibrium. Instead, the system shows periodic fluctuations in the densities of cooperators and defectors in the form of spatiotemporally traveling waves. During the dynamics, defectors exploit cooperators and advance in cooperators' domain. Once cooperators get extinct, defectors die out due to low resource availability. Cooperators, in turn, effectively escape from defectors by moving to new locations. Consequently, the dynamics exhibits traveling waves of cooperators chased by defectors. These waves annihilate each other upon clash.

In SV.3, $c = 0.1$ and $q = 0.1$. A snapshot of the densities of the individuals in this regime is presented in Supplementary Figure.6(c). In Supplementary Figure.7(c), the theta function of the densities, $\theta(\rho(x, y))$ is presented. In SV.4 $c = 1$ and $q = 0.1$. A snapshot of the densities of the individuals in this regime is presented in Supplementary Figure.6(d). In Supplementary Figure.7(d), the theta function of the densities, $\theta(\rho(x, y))$ is presented. In both cases, the dynamic is governed by traveling waves of cooperators and defectors. For a higher cost, SV.4, the system maintains a higher density of individuals arranged in compact moving fronts due to a higher rate of resource production in the game. Higher resource production, in turn, results from the formation of compact domains of cooperators in which cooperators can reap the benefit of cooperation among themselves.

Supplementary Note.4 Mean field equations

An example of the fixed point and periodic orbit of the mean-filed equation, derived in the Methods Section in the main text, are presented in Supplementary Figure.8. For a given λ, the mean filed equations settle in a fixed point, which can be one of the three equilibrium solutions, depending on the value of r. An example of the fixed point, for $r = 4$ is presented in Supplementary Figure.8, left panel. The mean-field equation predicts that the coexistence fixed point, III, becomes unstable at the critical value of r, and the dynamics show periodic fluctuations. An example of the periodic orbit predicted by the mean-field equation is presented in Supplementary Figure.8, right panel. The mean-field prediction of the critical enhancement factor above which non-equilibrium fluctuations set in is studied in the main text (figure 5, panel (c)). Quantitatively, the mean-field prediction of the onset of non-equilibrium fluctuations shows a small deviation from simulation results.

Supplementary Note.5 Phase transitions

While the transition to the evolution of cooperation is universal and independent of the parameter values, the onset of non-equilibrium dynamics can depend on the parameters and shows a strong sensitivity to c. This can be seen in Fig. Supplementary Figure.9(c), where the value of r above

which non-equilibrium fluctuations are observed is plotted. The prediction of the dynamical mean-field theory is also plotted by a dashed red line, which is in fair agreement with simulation results, especially for high costs. A simple argument suggests non-equilibrium fluctuations result from the fast growth of cooperators such that the system does not have enough time to settle in an economic equilibrium. Based on this argument, non-equilibrium fluctuations set in when the return to cooperation, $(r-1)c$, exceeds the resource consumption rate. Under this condition, solitary cooperators receive a net benefit and can grow exponentially fast. On the other hand, defectors can reap the benefit of cooperation only with a delay and when cooperators' density reaches a high value. Under exponential growth of cooperators, this time delay can destabilize the dynamics. This naive argument predicts non-equilibrium fluctuations occur at $r_n^* = 1 + \eta/c$. This curve is superimposed in Fig. Supplementary Figure.9(c), where it can be seen that it grasps the onset of non-equilibrium fluctuations fairly well, especially for small costs.

Finally, in Fig. Supplementary Figure.9(d), I study the onset of cooperation for zero resource generation rate. Here, γ as a function of r is plotted for different population structures and viscosity. $\frac{\eta}{(r-1)c-\eta}$ is also plotted. According to eq. **??**, these two should coincide. Noticeably, eq. **??** is derived from the resource conservation principle, which holds independently of population viscosity. As can be seen, the data from different population structures and population viscosity are well explained by the resource conservation equation, eq. **??**.

Supplementary Note.6 Life-history strategies

The lifespan distribution of cooperators for different population viscosity and population structures is presented in Fig. Supplementary Figure.10. In the equilibrium state, which occurs for small enhancement factors, the lifespan distribution shows an exponential decay (Fig. Supplementary Figure.10(a) to (d)). In a non-viscous population, the decay is faster for cooperators than for defectors. However, the lifespan distribution of cooperators and defectors shows less difference in a viscous population. Furthermore, the lifespan distribution of defectors shows a peak at young ages. This peak results from the fact that defectors rapidly die once they drive cooperators in their neighborhood into extinction. Finally, the lifespan distribution shows a faster decay for long ages in both viscous and structured populations. This signals that both viscosity and population structure favor a life history in which individuals reproduce more and die faster.

Lifespan distribution of cooperators and defectors in the non-equilibrium state for different population viscosity and population structures is presented in Fig. Supplementary Figure.10(e) to (h). In this case, the lifespan distributions show periodic decay. As we have seen before, the dynamics in this regime show periodic fluctuations, in which cycles of a high density of cooperators, followed by a high density of defectors, and then the decline of the population density is observed. The nth peak in the lifespan distributions corresponds to the individuals who survive for n cycles. Furthermore, the lifespan distributions show an exponentially decaying trend for long ages.

The mean lifespan of cooperators and defectors is studied in Supplementary Figure.11 and Supplementary Figure.12, for respectively, $\lambda = 0.6$ and$\lambda = 1$. In both cases, in a non-viscous population (panels (a) and (b)), defectors have a longer average lifespan. Both population viscosity and population structure decreases mean lifespan. Furthermore, population viscosity tends to decrease the difference between the mean lifespan of cooperators and defectors.

Supplementary Note.7 The densities of different strategies

The densities of individuals, ρ, fraction of cooperators, $m = \rho_C/\rho$, the density of cooperators, ρ_C, and the density of defectors, ρ_D, for a non-viscous, $q = 1$, and viscous $q = 0.1$ population, in $\lambda - r$ plane are color plotted, respectively, in Supplementary Figure.13 and Supplementary Figure.14. The phase boundary above which cooperation evolves is indicated in the figures by red circles. Different regions of the phase diagram, predicted by equilibrium solutions, are marked in Supplementary Figure.13. Here, a mixed population is considered. The situation is qualitatively similar for a structured population. In both cases, a defective phase where defectors survive at large resource regeneration rates, a coexistence region where cooperators and defectors coexist, and a purely cooperative phase where only cooperators survive is observed. Furthermore, in both cases, increasing the resource regeneration rate increases the density of defectors and decreases the fraction of cooperators.

In Supplementary Figure.15 and Supplementary Figure.16, per area birth rate of cooperators (a), defectors (b), and the per capita birth rate of cooperators (c) and defectors (d) are plotted. In both cases, the per-area birth rate is maximized for low resource regeneration rates and enhancement factors. This is due to the fact that in this region, the densities of individuals show strong periodic fluctuations. Furthermore, the per capita birth rate of cooperators is locally maximized on the phase transition between cooperative and defective phases.

To see the effect of population viscosity, in Supplementary Figure.17, I plot the difference between the densities of individuals, $\delta\rho = [\rho^{q=1} - \rho^{q=0.1}]/[\rho^{q=1} + \rho^{q=0.1}]$ (a), fraction of cooperators, $\delta m = [m^{q=1} - m^{q=0.1}]/[m^{q=1} + m^{q=0.1}]$, the densities of cooperators $\delta\rho_C = [\rho_C^{q=1} - \rho_C^{q=0.1}]/[\rho_C^{q=1} + \rho_C^{q=0.1}]$ (c), and the densities of defectors $\delta\rho = [\rho^{q=1} - \rho_D^{q=0.1}]/[\rho_D^{q=1} + \rho_D^{q=0.1}]$ (d), in a non-viscous and viscous population. Population viscosity has contrasting effects on population density. This difference reflects the effect of population viscosity on the game payoff of the individuals. Population viscosity slightly decreases the population density in the purely cooperative phase where defectors are found in the population only due to mutation. This is due to the fact that in this region, population viscosity can help mutant defectors to better exploit cooperators in their neighborhoods. This, in turn, has a detrimental effect on cooperation level, game payoff, and population density. For large resource regeneration rate and enhancement factors, population viscosity significantly decreases population density and the density and fraction of cooperators. This fact results from a shift in the cooperation-defection transition to lower resource regeneration rates in the presence of population viscosity. On the other hand, population viscosity facilitates cooperation for low resource regeneration rates and enhancement factors, where the density of individuals is lower.

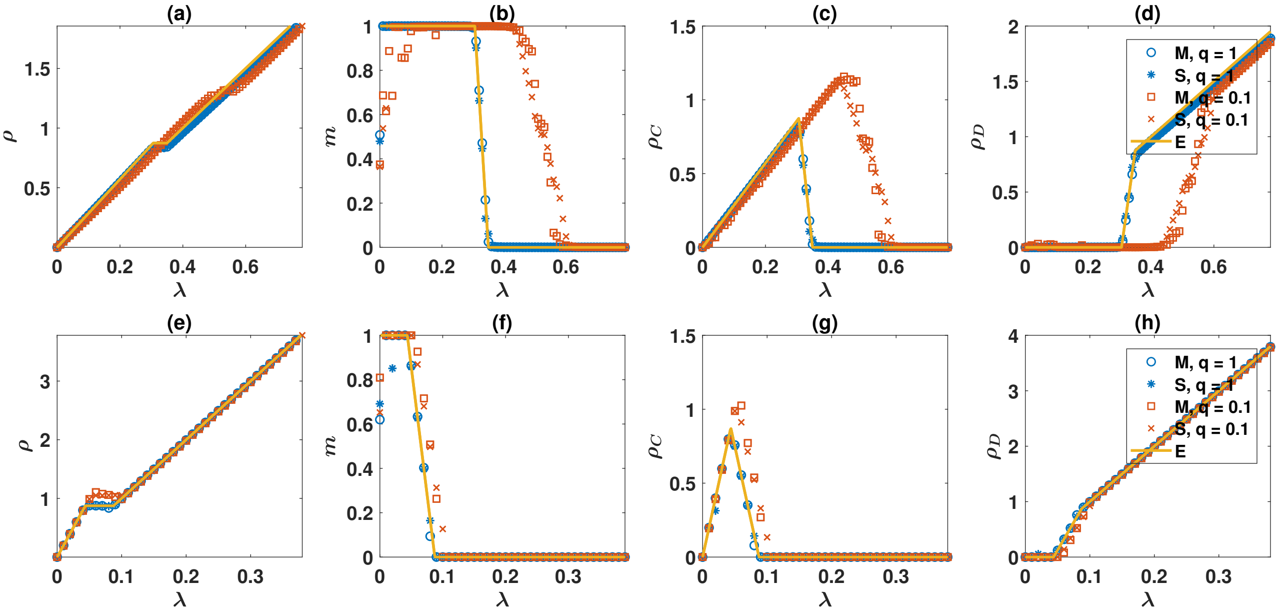

Supplementary Figure.1: The density, ρ (a) and (e), fraction of cooperators, m (b) and (f), the density of cooperators, ρ_C (c) and (g), and the density of defectors, ρ_D (d) and (h), as a function of the resource regeneration rate, λ, for $\eta = 0.4$, (a) to (d), and $\eta = 0.1$, (e) to (h). The equilibrium prediction, equation 7 in the main text, is shown by solid orange line. Cooperation evolves in poor environments and as the resource regeneration rate increases a phase transition to a defective phase is observed. Simulations in non-viscous populations in both mixed and structured populations (blue circle and stars) show a high agreement with equilibrium prediction up to the onset of non-equilibrium fluctuation for small λ. A small discrepancy between simulation results and equilibrium prediction is expected for large η due to negative internal resource of the deceasing individuals. Parameter values: $L = 100$, $\eta = 0.4$, $d = 2$, $r = 1.5$, $c = 0.1$, and $\nu = 10^{-4}$.

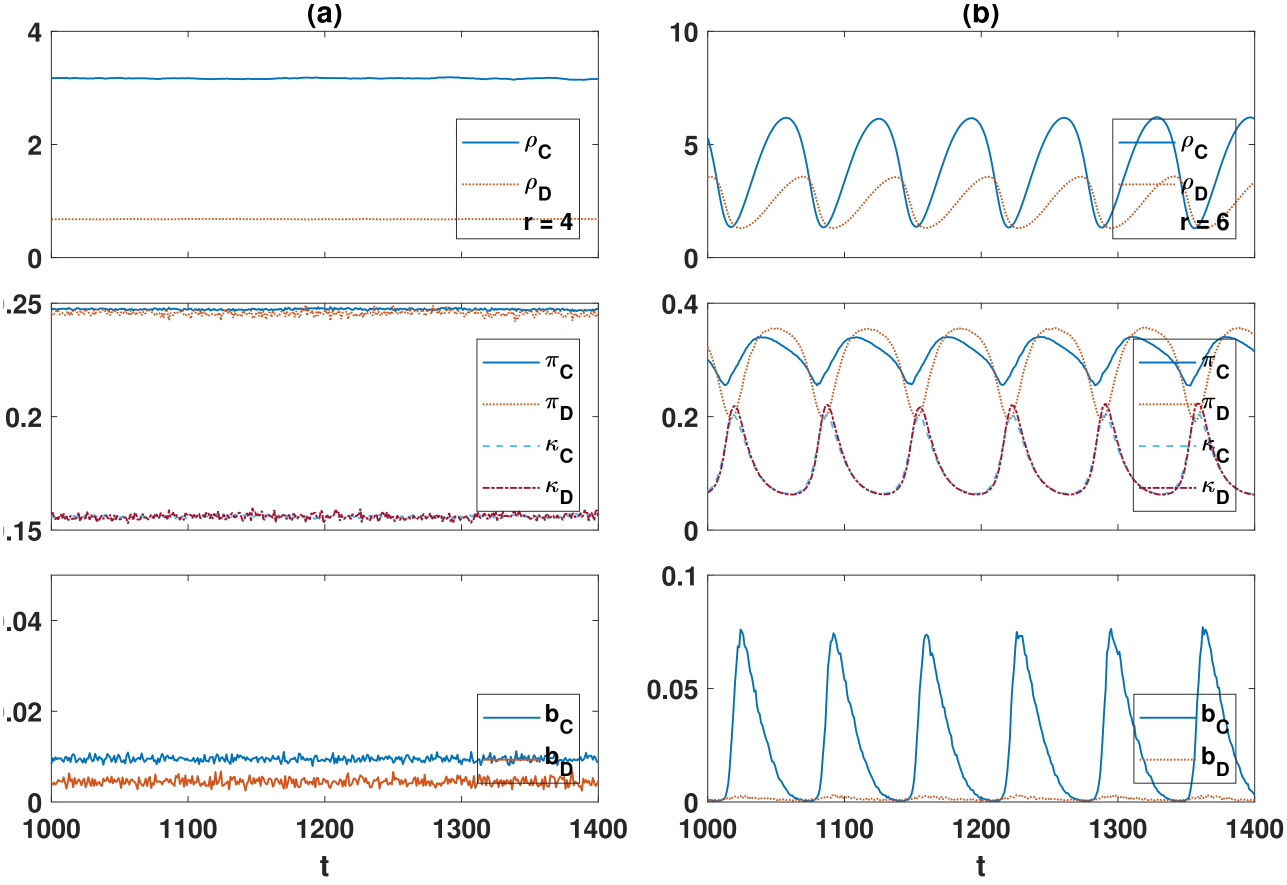

Supplementary Figure.2: Time evolution of the system in a non-viscous well-mixed population. Densities (top), payoffs (middle), and per capita birth rate (bottom) of cooperators and defectors in equilibrium (a) and non-equilibrium (b) states. The system can settle in a fixed point where payoffs of cooperators and defectors are equal and economic equilibrium holds (a), or remains in a non-equilibrium state where economic equilibrium is not reached, and densities and payoffs show periodic fluctuations (b). Cooperators show a higher population turnover (birth and death rate) than defectors in both states. Parameter values: $L = 100$, $\eta = 0.4$, $d = 2$, $\lambda = 0.6$, $c = 0.1$, $q = 1$, and $\nu = 10^{-4}$. A mixed population is used.

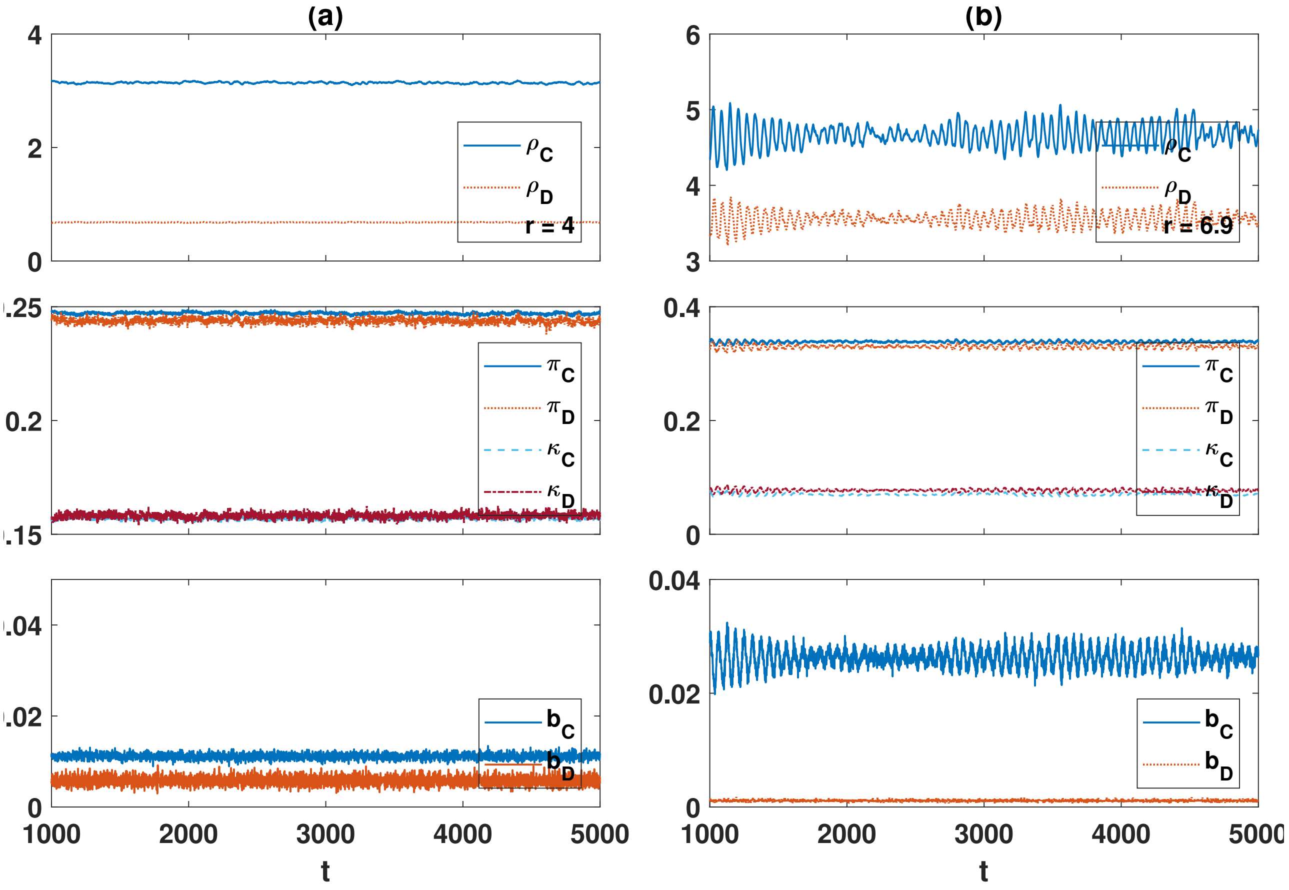

Supplementary Figure.3: Time evolution of the system in a non-viscous and structured population. Densities (top), payoffs (middle), and per capita birth rate (bottom) of cooperators and defectors in equilibrium (a) and non-equilibrium (b) states. The system can settle in a fixed point where payoffs of cooperators and defectors are equal and economic equilibrium holds (a), or remains in a non-equilibrium state where economic equilibrium is not reached, and densities and payoffs show periodic fluctuations (b). In structured populations, for sufficiently large system sizes, these fluctuations exhibit themselves in the form of traveling waves. In both states, cooperators show a higher population turnover (birth and death rate) than defectors. Parameter values: $L = 100$, $\eta = 0.4$, $d = 2$, $\lambda = 0.6$, $c = 0.1$, $q = 1$, and $\nu = 10^{-4}$.

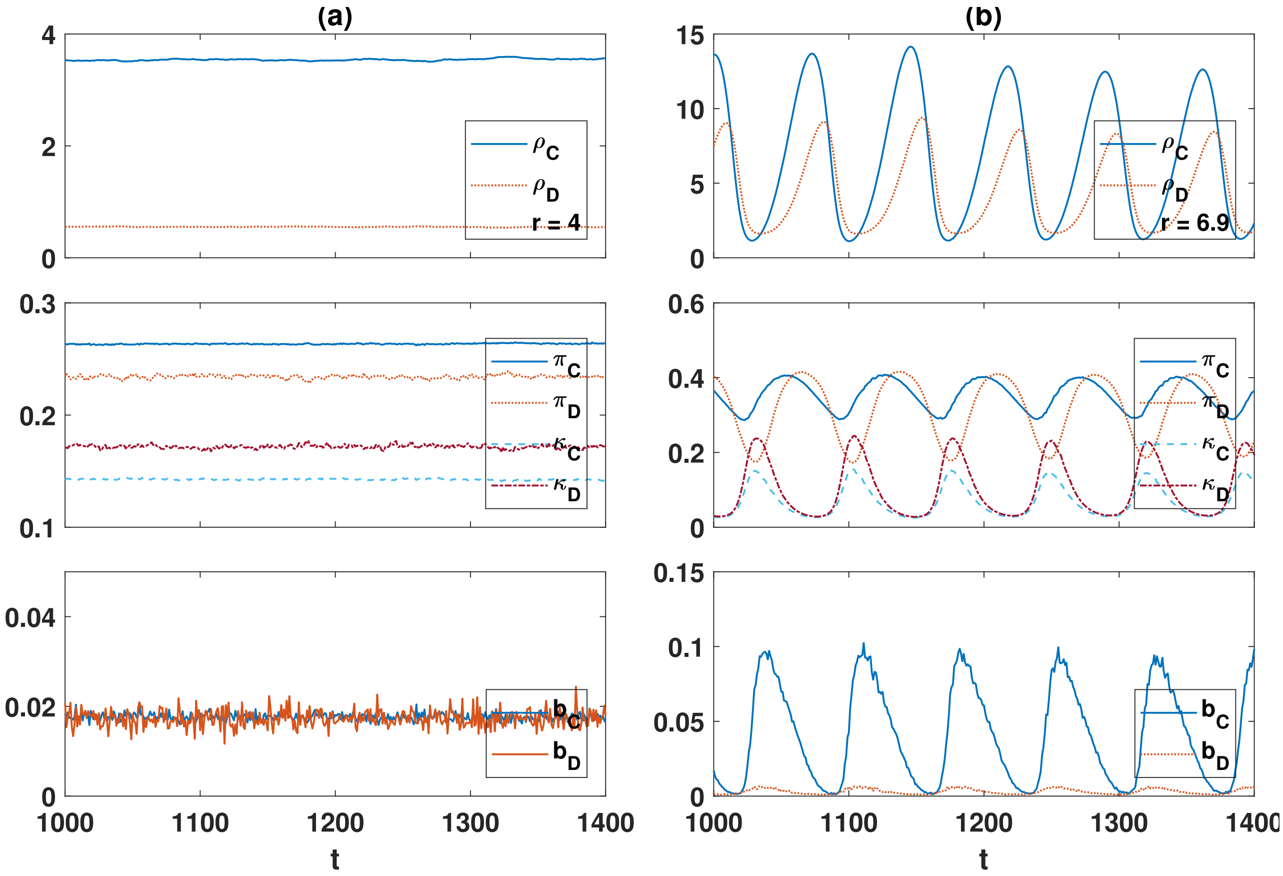

Supplementary Figure.4: Time evolution of the system in a viscous and mixed population. Densities (top), payoffs (middle), and per capita birth rate (bottom) of cooperators and defectors in equilibrium (a) and non-equilibrium (b) states. The system can settle in a fixed point where payoffs of cooperators and defectors are equal and economic equilibrium holds (a), or remains in a non-equilibrium state where economic equilibrium is not reached, and densities and payoffs show periodic fluctuations (b). In both states, cooperators receive a higher payoff than defectors from the game and have lower basal resource acquisition due to living in homogenous over-crowded domains. Population viscosity tends to diminish the population turnover of cooperators and defectors in the fixed point. However, cooperators have a higher population turnover than defectors in the non-equilibrium phase. Parameter values: $L = 100$, $\eta = 0.4$, $d = 2$, $\lambda = 0.6$, $c = 0.1$, $q = 1$, and $\nu = 10^{-4}$.

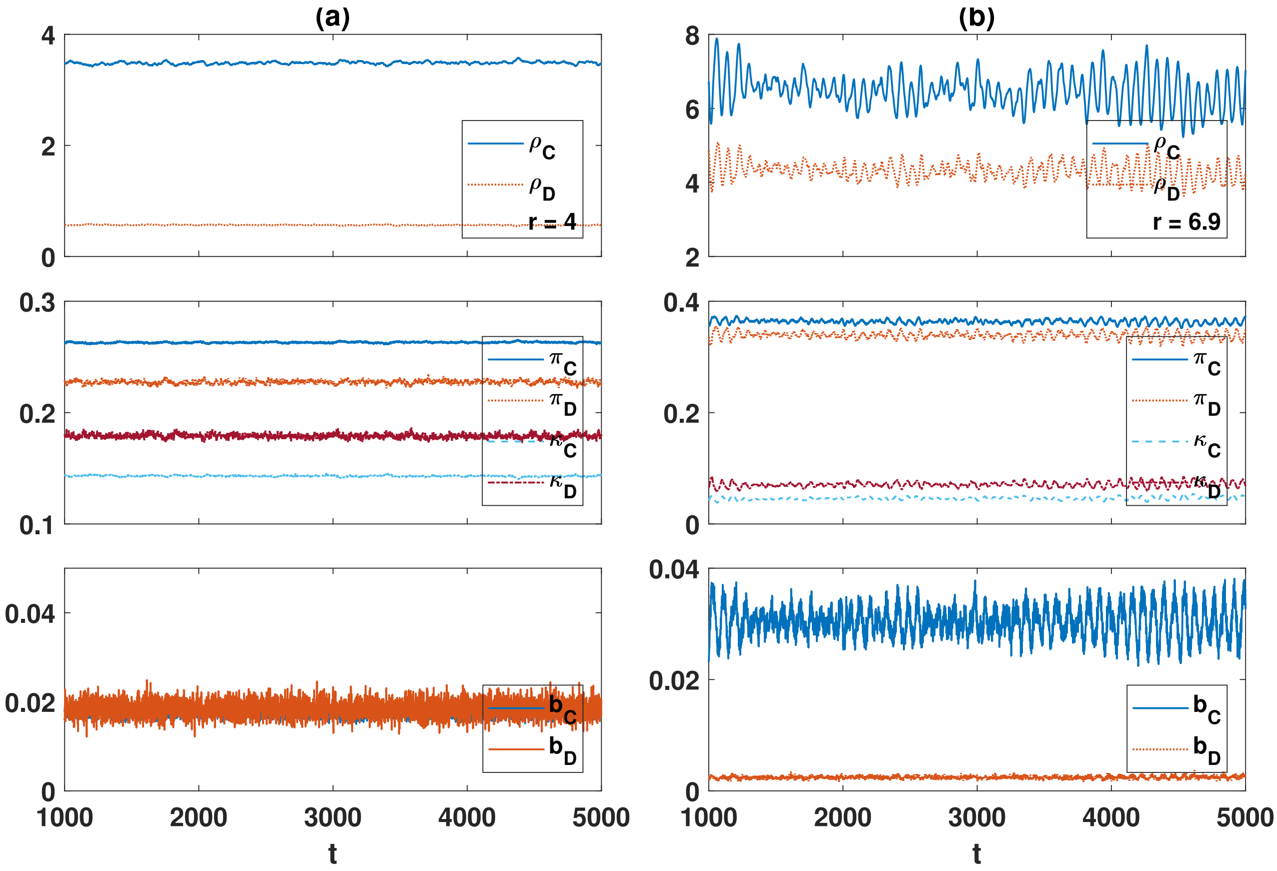

Supplementary Figure.5: Time evolution of the system in a viscous and structured population. Densities (top), payoffs (middle), and per capita birth rate (bottom) of cooperators and defectors in equilibrium (a) and non-equilibrium (b) states. The system can settle in a fixed point where payoffs of cooperators and defectors are equal and economic equilibrium holds (a), or remains in a non-equilibrium state where economic equilibrium is not reached, and densities and payoffs show periodic fluctuations (b). In structured populations, for sufficiently large system sizes, these fluctuations exhibit themselves in the form of traveling waves. In both states, cooperators receive a higher payoff than defectors from the game and have lower basal resource acquisition due to living in homogenous over-crowded domains. Population viscosity tends to diminish the population turnover of cooperators and defectors in the fixed point. However, cooperators have a higher population turnover than defectors in the non-equilibrium phase. Parameter values: $L = 100$, $\eta = 0.4$, $d = 2$, $\lambda = 0.6$, $c = 0.1$, $q = 1$, and $\nu = 10^{-4}$.

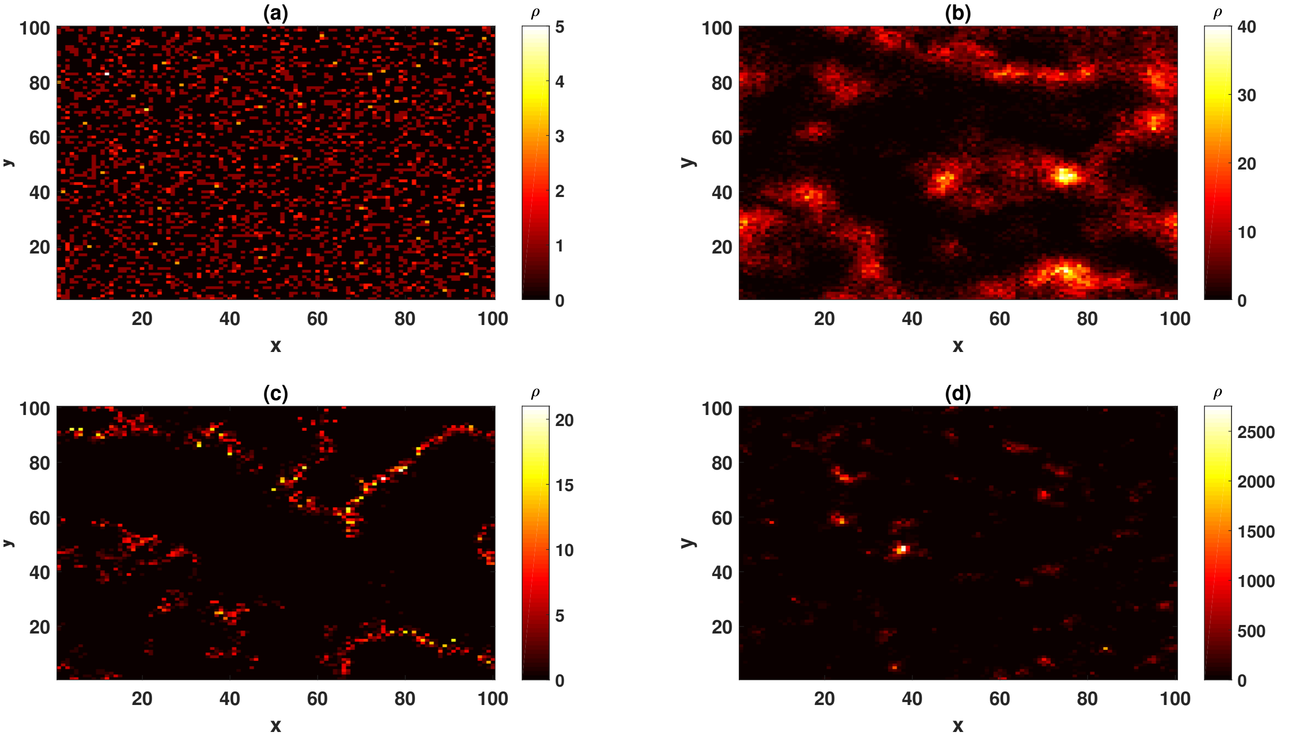

Supplementary Figure.6: Snapshots of the time evolution of the system. The densities of the individuals in the stationary state. Parameter values: $d = 2$, $\nu = 10^{-2}$, $\eta = 0.4$, $r = 2$, $\lambda = 0.1$, $L = 100$. In (a) $c = 0.1$ and $q = 1$, in (b) $c = 1$ and $q = 1$, in (c) $c = 0.1$ and $q = 0.1$, and in (d) $c = 1$ and $q = 0.1$.

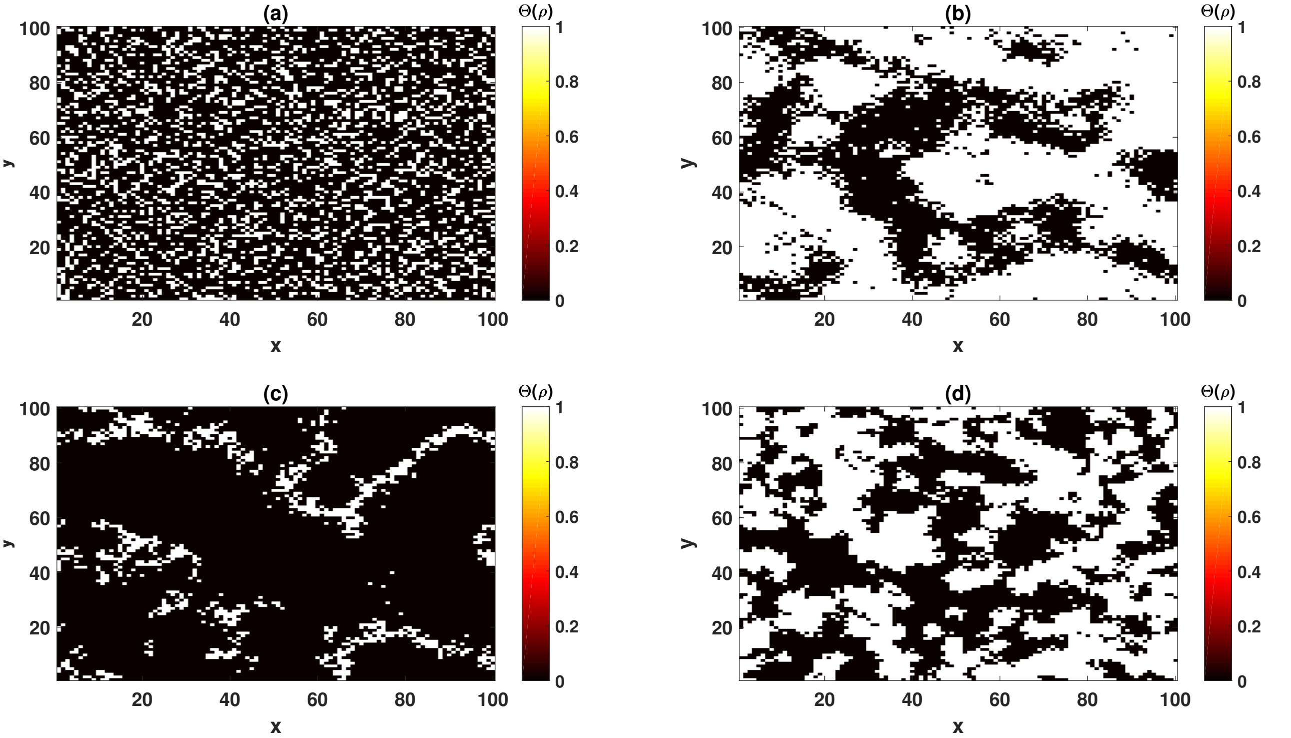

Supplementary Figure.7: Snapshots of the time evolution of the system. The theta function of densities of the individuals, $\Theta(\rho(x,y))$, which is one for nonzero densities and zero otherwise, in the stationary state. Parameter values: $d=2$, $\nu=10^{-2}$, $\eta=0.4$, $r=2$, $\lambda=0.1$, $L=100$. In (a) $c=0.1$ and $q=1$, in (b) $c=1$ and $q=1$, in (c) $c=0.1$ and $q=0.1$, and in (d) $c=1$ and $q=0.1$.

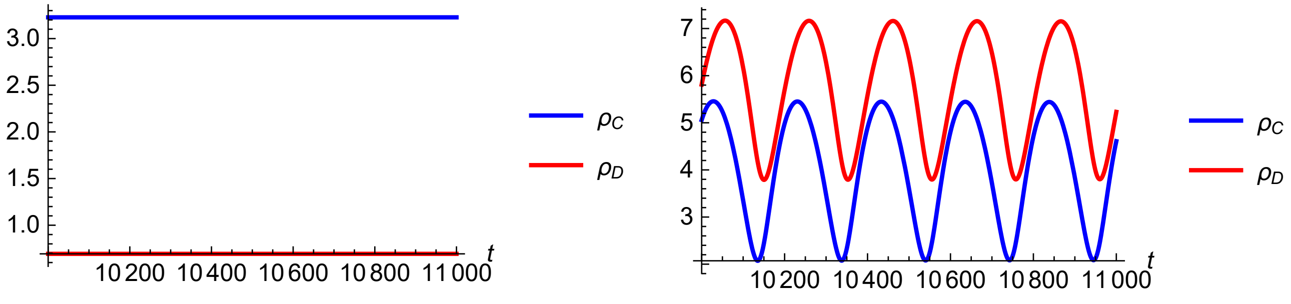

Supplementary Figure.8: Time evolution of the mean-field equations. Densities of cooperators, ρ_C, and defectors, ρ_D, in equilibrium (left) and non-equilibrium (right) states. Consistently with simulations, the mean-field equations predict the dynamics settle in a fixed point for small enhancement factors (left $r=4$). As the enhancement factors increases ($r=9.2$ right), the fixed point becomes unstable and periodic fluctuations sets in. Parameter values: $\eta=0.4$, $\lambda=0.6$, $c=0.1$, and $\nu=10^{-4}$.

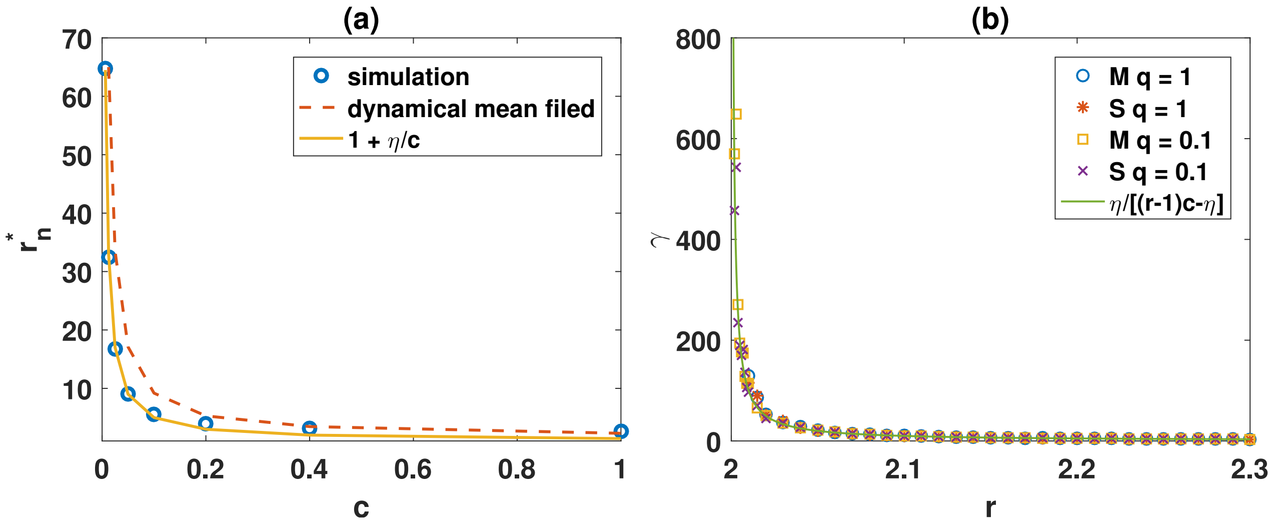

Supplementary Figure.9: Phase transitions. (a): The onset of non-equilibrium fluctuations determined from simulations (blue circles), dynamical mean-field equation, derived in the Methods in the main text (dashed red), and a naive argument based on the exponential growth of cooperators (solid orange). (b): The ratio of cooperators to defectors, γ, close to the onset of cooperation for zero resource regeneration rate. Markers show simulations for mixed and structured, and viscous and non-viscous populations, and the solid green line shows the prediction of the resource conservation principle. In (c) $\eta = 0.4$, $\lambda = 0.6$, $q = 1$, and a mixed population is used, and in (d), $\eta = 0.2$, $c = 0.1$, and $\lambda = 0$. In (c) and (d) $L = 100$, $d = 2$, and $\nu = 10^{-4}$.

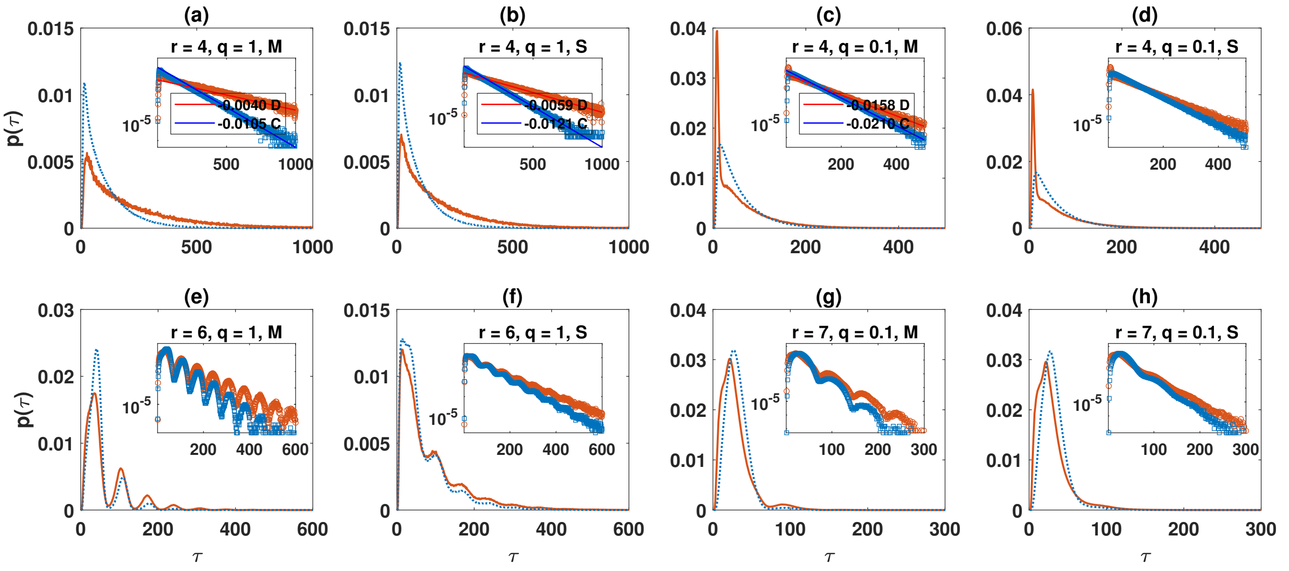

Supplementary Figure.10: The lifespan distribution. The lifespan distribution of cooperators (dotted blue) and defectors (solid red) for different population structures and viscosity (indicated in the figure). Insets show lifespan distributions in a semi-logarithmic plot. Lifespan distributions show a characteristic age with an exponential cut-off at long ages, which is faster for cooperators. In a viscous population, the lifespan distribution of defectors shows a peak at young ages. In (a) to (d), the system settles in the fixed point, and in (e) to (h), nonequilibrium fluctuations are observed. In the nonequilibrium state, lifespan distributions show periodic decay for long ages. The nth peak corresponds to individuals who survive for n cycles. Parameter values: $L = 100$, $\eta = 0.4$, $d = 2$, $\lambda = 0.6$, $c = 0.1$, and $\nu = 10^{-4}$.

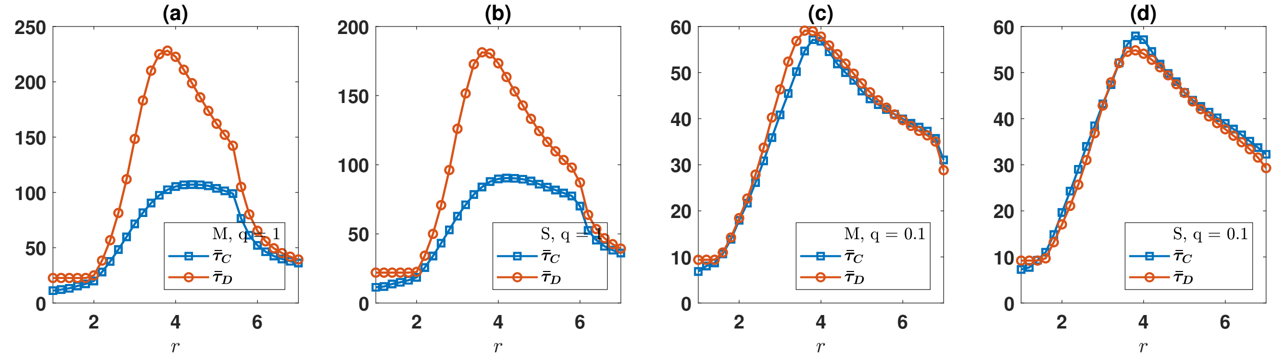

Supplementary Figure.11: Mean lifespan of cooperators and defectors for $\lambda = 0.6$. The mean lifespan of cooperators (blue squares) and defectors (red circles) for a non-viscous ($q = 1$), and viscous ($q = 0.1$), and structured (S) and mixed (M) populations as a function of the enhancement factor, r is shown. In a non-viscous population, defectors age more than cooperators, on average. The onset of non-equilibrium fluctuations marks a decline in the mean lifespan of the individuals. Both the population structure and population viscosity decrease the lifespan of the individuals. Furthermore, population viscosity tends to diminish the age differences of cooperators and defectors. Parameter values: $L = 100$, $\eta = 0.4$, $d = 2$, $\lambda = 0.6$, $c = 0.1$, and $\nu = 10^{-4}$.

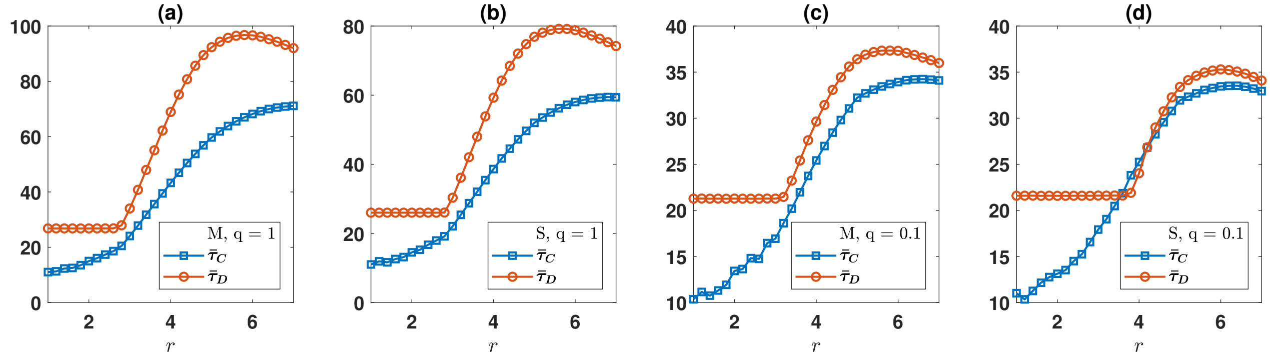

Supplementary Figure.12: Mean lifespan of cooperators and defectors for $\lambda = 1$. The mean lifespan of cooperators (blue squares) and defectors (red circles) for a non-viscous ($q = 1$), and viscous ($q = 0.1$), and structured (S) and mixed (M) populations as a function of the enhancement factor, r is shown. In a non-viscous population, defectors age more than cooperators, on average. The onset of non-equilibrium fluctuations marks a decline in the mean lifespan of the individuals. Both the population structure and population viscosity decrease the lifespan of the individuals. Furthermore, population viscosity tends to diminish the age differences of cooperators and defectors. Parameter values: $L = 100$, $\eta = 0.4$, $d = 2$, $\lambda = 1$, $c = 0.1$, and $\nu = 10^{-4}$.

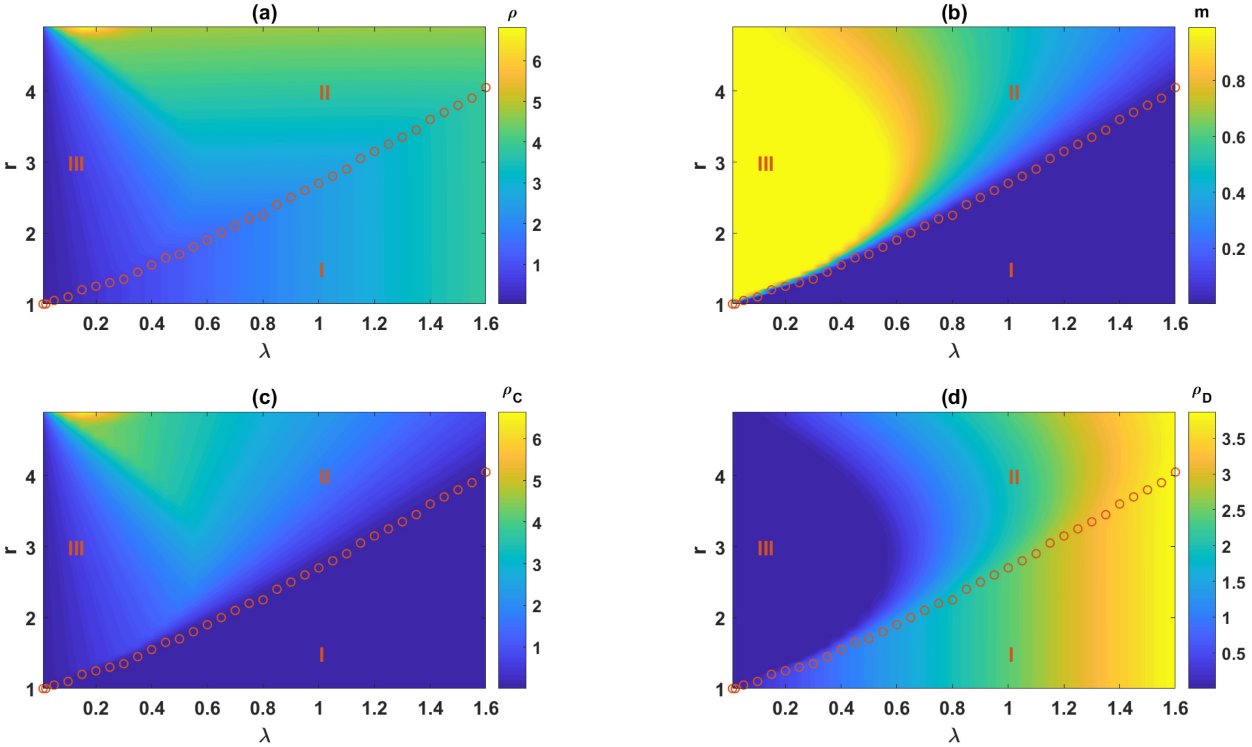

Supplementary Figure.13: The density of different strategies. The density of individuals, ρ (a), fraction of cooperators, m (b), the density of cooperators, ρ_C (c), and the density of defectors, ρ_D (d), in a non-viscous and well-mixed population. The three regimes, defective phase in which cooperation does not evolve in small enhancement factors (I), cooperative phase with zero density of defectors (III) for large enhancement factors, and coexistence region, where cooperators and defectors coexist (II) for large enhancement factors and resource regeneration rates, are marked. The phase transition line above which cooperation evolves is marked by red circles. Parameter values: $L = 100$, $d = 2$, $\nu = 10^{-3}$, $c = 0.1$, $q = 1$. The simulations are run for 5000 time steps, and an average over the last 1000 steps is taken.

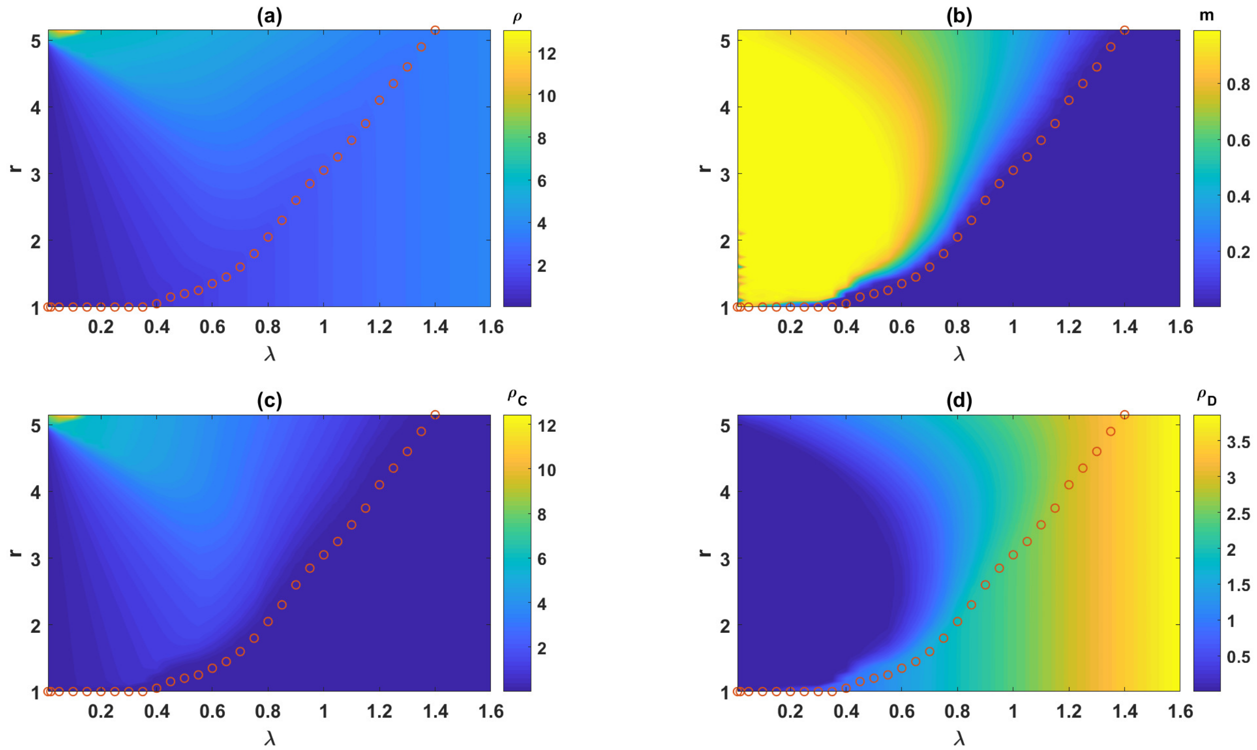

Supplementary Figure.14: The density of different strategies. The density of individuals, ρ (a), fraction of cooperators, m (b), the density of cooperators, ρ_C (c), and the density of defectors, ρ_D (d), in a viscous and well-mixed population. The three regimes, defective phase in which cooperation does not evolve in small enhancement factors, cooperative phase with zero density of defectors for large enhancement factors, and coexistence region, where cooperators and defectors coexist for large enhancement factors and resource regeneration rates, are distinguishable. The phase transition line above which cooperation evolves is marked by red circles. Parameter values: $L = 100$, $d = 2$, $\nu = 10^{-3}$, $c = 0.1$, $q = 0.1$. The simulations are run for 5000 time steps, and an average over the last 1000 steps is taken.

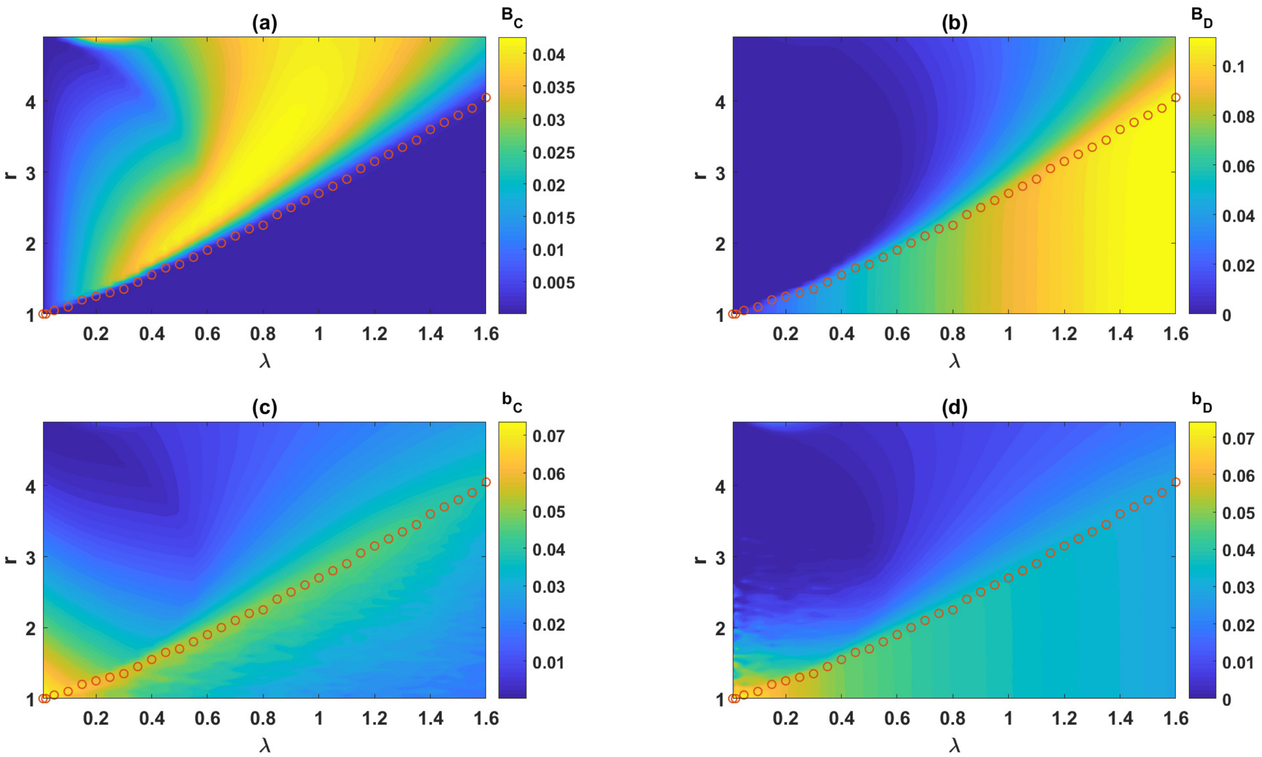

Supplementary Figure.15: Reproduction of cooperators and defectors in a non-viscous population. Per area birth rate of cooperators, B_C (a), per area birth rate of defectors, B_D (b), per capita birth rate of cooperators, b_C (c), per capita birth rate of defectors, b_D (d), in a non-viscous and well-mixed population are shown. As the population is in a stationary state, the death rates are similar. Cooperators show a higher per capita birth rate and population turnover in the region where they survive. The population turnover of cooperators is maximized on the cooperation-defection transition. Parameter values: $L = 100$, $d = 2$, $\nu = 10^{-3}$, $c = 0.1$, $q = 1$. The simulations are run for 5000 time steps, and an average over the last 1000 steps is taken.

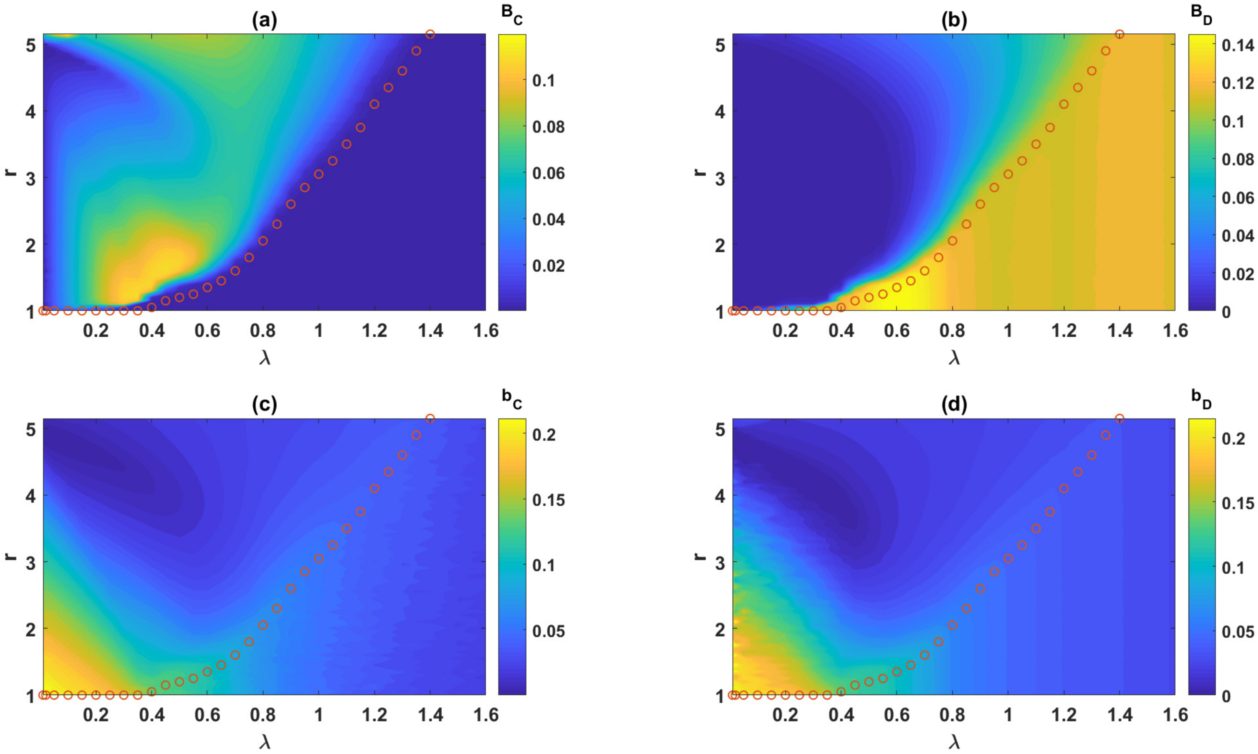

Supplementary Figure.16: Reproduction of cooperators and defectors in a viscous population. Per area birth rate of cooperators, B_C (a), per area birth rate of defectors, B_D (b), per capita birth rate of cooperators, b_C (c), per capita birth rate of defectors, b_D (d), in a viscous and well-mixed population. As the population is in a stationary state, the death rates are similar. Cooperators show a higher per capita birth rate and population turnover in the region where they survive. The population turnover of cooperators is maximized on the cooperation-defection transition. The Parameter values: $L = 100$, $d = 2$, $\nu = 10^{-3}$, $c = 0.1$, $q = 0.1$. The simulations are run for 5000 time steps, and an average over the last 1000 steps is taken.

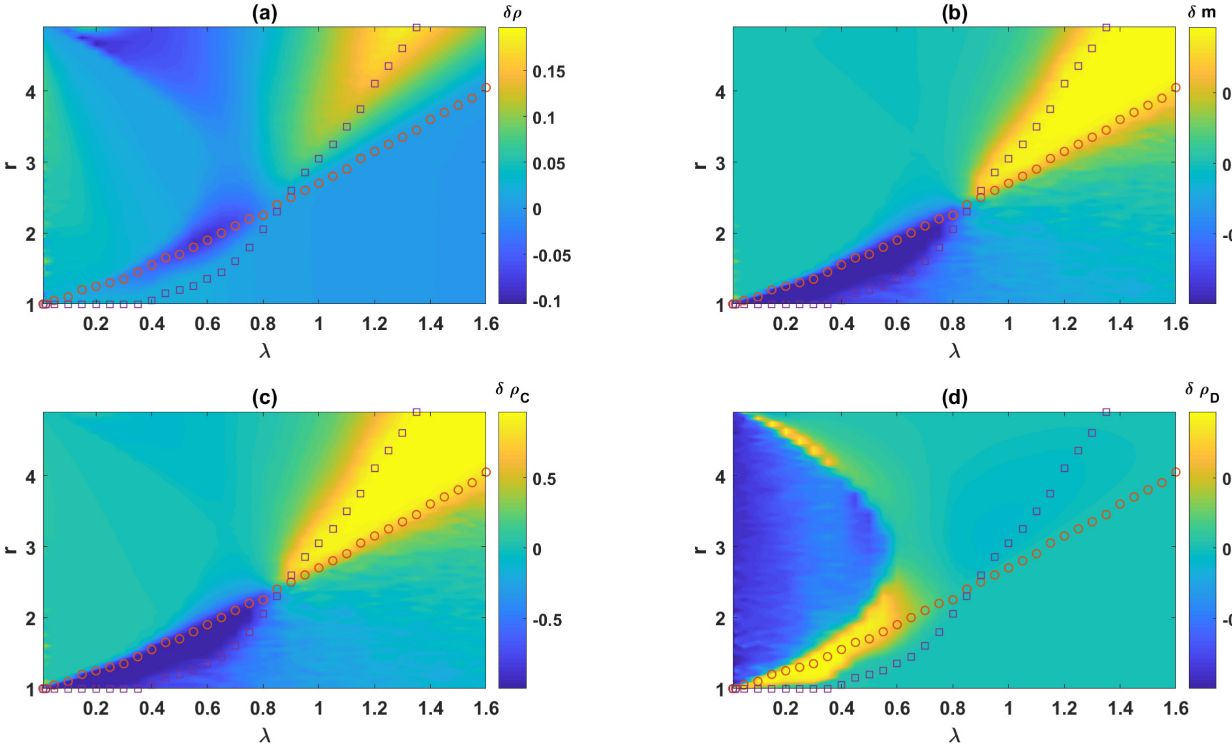

Supplementary Figure.17: The effect of population viscosity. The difference between the densities of individuals, $\delta\rho = [\rho^{q=1} - \rho^{q=0.1}]/[\rho^{q=1} + \rho^{q=0.1}]$ (a), fraction of cooperators, $\delta m = [m^{q=1} - m^{q=0.1}]/[m^{q=1} + m^{q=0.1}]$, the densities of cooperators $\delta\rho_C = [\rho_C^{q=1} - \rho_C^{q=0.1}]/[\rho_C^{q=1} + \rho_C^{q=0.1}]$ (c), and the densities of defectors $\delta\rho = [\rho^{q=1} - \rho_D^{q=0.1}]/[\rho_D^{q=1} + \rho_D^{q=0.1}]$ (d), in a non-viscous and viscous population. Population viscosity can be beneficial for the evolution of cooperation in poor environments (small λ), but detrimental in rich environments. Parameter values: $d = 2$, $\nu = 10^{-3}$, $c = 0.1$, $L = 100$. A mixed population is used. The simulations is run for 5000 time steps and an average over the last 1000 steps is taken.
